# Parametric analysis of colony morphology of non-labelled live human pluripotent stem cells for cell quality control

**DOI:** 10.1038/srep34009

**Published:** 2016-09-26

**Authors:** Ryuji Kato, Megumi Matsumoto, Hiroto Sasaki, Risako Joto, Mai Okada, Yurika Ikeda, Kei Kanie, Mika Suga, Masaki Kinehara, Kana Yanagihara, Yujung Liu, Kozue Uchio-Yamada, Takayuki Fukuda, Hiroaki Kii, Takayuki Uozumi, Hiroyuki Honda, Yasujiro Kiyota, Miho K Furue

**Affiliations:** 1 Department of Basic Medicinal Sciences, Graduate School of Pharmaceutical Sciences, Nagoya University, Furocho, Chikusa-ku, Nagoya 464-8601, Japan; 2 Stem Cell Evaluation Technology Research Center (SCETRA), Hacho-bori, Chuou-ku, Tokyo 104-0032, Japan; 3 Department of Biotechnology, Graduate School of Engineering, Nagoya University, Furocho, Chikusa-ku, Nagoya 464-8602, Japan; 4 Laboratory of Stem Cell Cultures, National Institutes of Biomedical Innovation, Health and Nutrition, Ibaraki, Osaka 567-0085, Japan; 5 Department of Cellular and Molecular Biology, Basic Life Sciences, Institute of Biomedical & Health Sciences, Hiroshima University, Hiroshima 734-8553, Japan; 6 Laboratory of Animal Models for Human Diseases, National Institutes of Biomedical Innovation, Health and Nutrition, Ibaraki, Osaka 567-0085, Japan; 7 Nikon Corporation, Nagaodaicho, Yokohama, Kanagawa 244-8533, Japan

## Abstract

Given the difficulties inherent in maintaining human pluripotent stem cells (hPSCs) in a healthy state, hPSCs should be routinely characterized using several established standard criteria during expansion for research or therapeutic purposes. hPSC colony morphology is typically considered an important criterion, but it is not evaluated quantitatively. Thus, we designed an unbiased method to evaluate hPSC colony morphology. This method involves a combination of automated non-labelled live-cell imaging and the implementation of morphological colony analysis algorithms with multiple parameters. To validate the utility of the quantitative evaluation method, a parent cell line exhibiting typical embryonic stem cell (ESC)-like morphology and an aberrant hPSC subclone demonstrating unusual colony morphology were used as models. According to statistical colony classification based on morphological parameters, colonies containing readily discernible areas of differentiation constituted a major classification cluster and were distinguishable from typical ESC-like colonies; similar results were obtained via classification based on global gene expression profiles. Thus, the morphological features of hPSC colonies are closely associated with cellular characteristics. Our quantitative evaluation method provides a biological definition of ‘hPSC colony morphology’, permits the non-invasive monitoring of hPSC conditions and is particularly useful for detecting variations in hPSC heterogeneity.

Human pluripotent stem cells (hPSCs), such as human embryonic stem cells (hESCs)^1^ and human induced pluripotent stem cells (hiPSCs)^2^,^3^, demonstrate high variability resulting from genomic variations and differences in methylation status, transcription, cell signalling and culture methods. The utility of hPSCs is further limited by the cellular phenotypic changes that are frequently observed following prolonged culture^4^,^5^,^6^,^7^,^8^,^9^,^10^. Therefore, the routine characterization of hPSCs using several standard criteria^11^,^12^, such as cell growth, marker expression, karyotype analysis and *in vitro* differentiation, is required to confirm hPSC status and viability. Colony morphology is one such criterion that is used to continuously evaluate hPSC health. Typical healthy undifferentiated hPSCs appear as tightly packed, round cells with large nuclei and notable nucleoli without spaces between cells^13^. The morphology of unhealthy hPSCs differs from that of normal hPSCs. However, manual evaluation of colony morphology is not quantitative. In several studies, morphology has been correlated with hPSC quality^13^,^14^,^15^,^16^,^17^, but the majority of these measurement techniques are based on fluorescent labelling via immunostaining or gene transfection. Further, hPSCs that have undergone immunostaining or gene expression analysis are not suitable for further research experiments.

Recently, image analysis combined with computational data processing has facilitated the evaluation of cellular status based on non-labelled images^17^,^18^,^19^,^20^,^21^,^22^,^23^,^24^,^25^. Machine learning, which involves pattern recognition and computational learning theory, is one of the most widely used strategies. Tokunaga *et al.*^18^ reported the utility of image-based computational classification to distinguish completely reprogrammed induced pluripotent stem cells (iPSCs) from incompletely reprogrammed cells. Cell morphology changes dynamically during iPSC reprogramming of somatic cells, resulting in many colonies with various morphology types in subsequent cultures. At present, iPSC colonies are manually selected based on morphology. However, the selection of completely reprogrammed iPSC colonies is technically challenging. Utilizing representative iPSC colony images of incompletely reprogrammed iPSCs selected by experts, it is possible to train computers to classify colonies according to morphological patterns. Previously, we reported an image-based quality evaluation method to predict the differentiation potential of human mesenchymal stem cells (hMSCs)^21^,^22^,^23^,^24^. The rates of 3 types of differentiation potential (osteogenic, chondrogenic and adipogenic) after one month of differentiation culture could be predicted based on multiple morphological parameters measured from cellular images obtained during the first 4 days of expansion^24^. The technical combination of ‘massive and stable non-biased images of cells’ and ‘multi-parametric analysis’ permits the analysis of cellular morphological features and replaces analyses dependent on conventional cell biological features such as marker profiles or gene expression determined via invasive methods. The combination of hardware, software and experimental design, particularly the incorporation of detailed biological data as an analytical foundation, facilitates the successful prediction of the biological characteristics of hMSCs by evaluating their cell morphology. Thus, non-invasive computation image analysis possesses utility in the quantitative morphological analysis of hPSC quality.

Here, we propose a non-invasive image-based evaluation method for detecting partially differentiated colony morphology in heterogeneous colony populations via live image analysis. A parent cell line exhibiting typical embryonic stem cell (ESC)-like morphology and an aberrant hPSC subclone (#12 trisomy) exhibiting partially differentiated colony morphology were used as models to validate the utility of our quantitative evaluation method. We then analysed global gene expression profiles in individual colonies. Colony morphology classification based on the statistical analysis of non-labelled live-cell images with unbiased morphological parameters was compared with classification based on global gene expression profiles of individual colonies. Classification utilizing statistical analysis produced similar results compared to classification utilizing gene expression profiles. Thus, our quantitative morphological evaluation method facilitates the non-invasive analysis of hiPSC conditions and demonstrates particular utility in monitoring variations in hPSC heterogeneity.

## Results

### Construction of a colony database

In the present study, we examined the morphologies of an aberrant 201B7-1A subclone that gained an additional copy of chromosome 12 after prolonged culture and its healthy parent 201B7 line, which was the first hiPSC line reported by Yamanaka’s group^2^ (Supplementary Tables S1–3 and Supplementary Fig. S1). The 201B7-1A subclone possesses an antigenic profile that is similar to the 201B7 cell line and is characterised by the undifferentiated cell surface markers Tra-1-60, Tra-1-81, Tra-2-54, human stage-specific embryonic antigen (SSEA) 3, SSEA4 and CD90 (Supplementary Fig. S1B) and the ability to differentiate into 3 germ layers, confirmed by teratoma and embryoid body formation assays (Supplementary Fig. S1 and Supplementary Table S2). However, the 201B7-1A subclone frequently exhibits unusual undifferentiated ESC-like colony morphology with irregularly collapsed colony edges and unclear borders, whereas the 201B7 clone exhibits typical ESC-like colony morphology (Supplementary Fig. S1A and Supplementary Videos S1 and S2). In addition, to incorporate a wider range of morphologies into the colony database, we included images of the 253G1 cell line^3^, which was established by Yamanaka’s group through the use of Yamanaka factors without *C-MYC*, and its subclone 253G1-B1, which tends to gain an additional copy of chromosome 12 (Supplementary Tables S1–3 and Supplementary Fig. S1).

First, a colony image library was constructed using the 4 hiPSC clones described above (schematic flow illustrated in Supplementary Fig. S2, Step 1). To eliminate bias from conventional microscopic cell image data, the optical conditions of the cell culture observation system were adjusted to collect stable and large-scale images with minimal imaging bias, which is manually derived from lighting, focusing, view-field selection and time-point scheduling (Supplementary Fig. S3). To include a range of colony morphologies during growth, 384 images (64 images × 6 wells) were acquired over 6 days of culture at 8-h intervals (Supplementary Fig. S2, Step 1-1). From the colony image library, which consisted of 2,304 images covering 999 individually recognised colonies, 303 colonies cultured to >1 mm in diameter were selected for further analysis. Smaller colonies were not included in the analysis due to larger variations in observed morphologies during subsequent culture. Then, 120 colony morphology parameters in the database were evaluated (Supplementary Fig. S2, Step 1–2). Highly correlated parameters (Pearson’s correlation coefficient: >0.98) and parameters that inadequately represented morphological characteristics (coefficient of variation: >30) were eliminated as these parameters had no utility in colony classification. Then, 8 major categories of morphological parameters comprising 27 individual parameters were used to quantitatively describe individual colonies (Supplementary Table S4).

**Figure 1:**
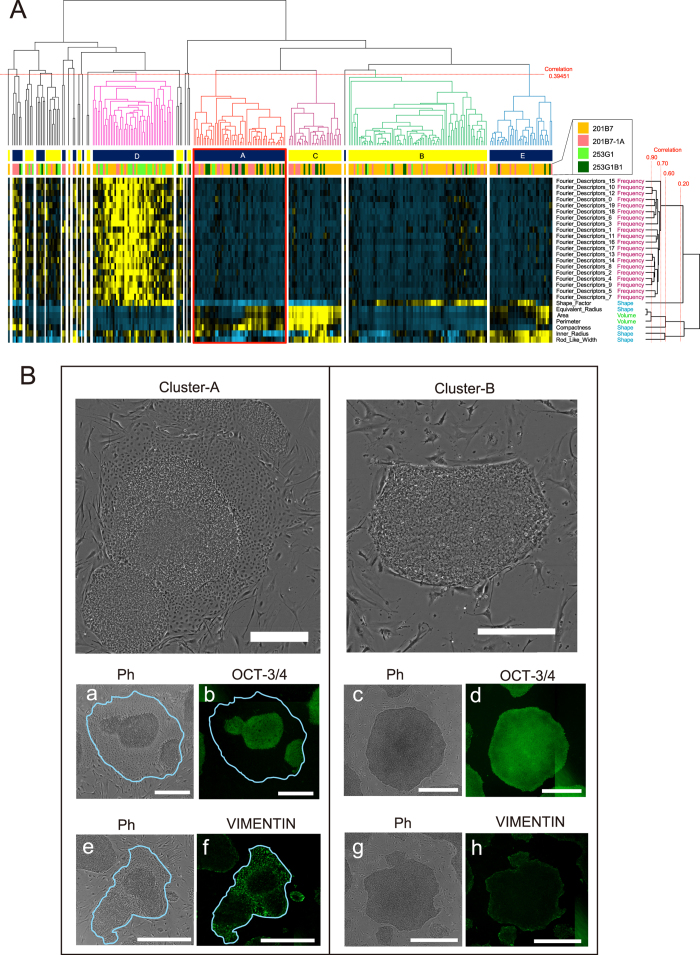
Overview of the morphological varieties and classified colonies of 4 hiPSC clones. (**A**) Clustering of colony morphologies in the colony database according to morphological parameters. Horizontal branches show the hierarchical clustering results divided into 20 clusters at a threshold of 0.39451. Major clusters (**A**–**E**) are indicated with coloured branches. Red branches indicate colonies categorised into cluster-A, the irregular colony morphology cluster. Vertical branches show the correlation of measured morphological parameters categorized into 3 types (frequency, shape and volume). Based on the heat map colour gradients (blue: low; yellow: high), cluster-A can be described as the combination of a relatively mature morphology (high in volume parameters), with a boundary that is not round (low in shape parameters), and a very irregular morphology (low in frequency). Morphological interpretations of each cluster are described in Supplementary Table S5. (**B**) Representative images of the colonies in cluster-A/cluster-B. Left upper image, a and e, cluster-A colonies exhibit disrupted peripheral colony edges. The tightly packed colonies were partially disrupted by fibroblastic cellular morphology. The sky-blue overlay mask indicates the colony area recognised by our image analysis. Right upper image, c and g, colonies classified as cluster-B are typical ES-like growing colonies. Fixed colonies were immunohistochemically stained with an anti-OCT-3/4 b and d antibody and an anti-VIMENTIN antibody (f and h). Given that 8 × 8 tiling images were merged to form an image in our analysis, partially bright and biased fluorescent areas are occasionally observed in the images, derived from the edges of individual images. Scale bar = 500 μm.

Second, colony morphology in the database was analysed by performing hierarchical clustering using the open source clustering software Cluster 3.0 and sorting colonies into morphological categories depending on the patterns exhibited by multiple parameters (Supplemetary Fig. S2, Step 1–3). The following clusters comprising >5% of total colonies in the database were defined as ‘major clusters’: cluster-A, cluster-B, cluster-C, cluster-D and cluster-E (Fig. 1A and Supplementary Table S5). This analysis eliminated arbitrary interference by selecting specific thresholds, parameters or categories and automatically determining ‘commonly observed morphology’. The proportion of 201B7-1A colonies categorised into cluster-A (27.3%) was higher than that of 201B7 colonies (13.3%), whereas the proportions of 201B7-1A colonies categorised into the other major categories were lower or approximately equal to those of 201B7 colonies. The proportion of 253G1-B1 colonies in cluster-A (40.5%) was higher than that of 253G1 colonies (4.8%). The actual morphologies of the representative clusters were manually verified (Fig. 1B, Supplementary Fig. S4 and Supplementary Table S6). Cluster-A colonies were characterised by the loss of clear colony edges, a comparatively flatter cytoplasm and a low nucleus-to-cytoplasm ratio. Although the colonies in cluster-B and cluster-D were comparatively smaller or partially surrounded by flatter cells, they exhibited a central area with hESC-like morphology. The morphologies of colonies in cluster-C and cluster-E resembled typical hESC-like morphologies of growing colonies. Thus, the morphology of 201B7-1A colonies in cluster-A may constitute a major signature morphology that is distinguishable from 201B7 colony morphology.

To evaluate the accuracy of classification based on colony morphology, the labels of 201B7 and 253G1 colonies and their subclones in the colony database were masked, and the colonies were again classified using the same analytical procedure. The colonies were classified in the same manner as used for the above classification (Supplementary Fig. S5). Then, the plate was physically rotated 180° and analysed again to measure morphological parameters. Because the placement of the culture vessel was originally adjusted for parameter measurement, measuring in the opposite orientation decreased the image quality and focus. Consequently, the parameter values changed slightly (Table S7). Among 15 colonies recognised on the plate, whereas 14 colonies were classified into the same clusters, one colony was classified into a different cluster, shown in Supplementary Fig. S6. Analysis revealed the presence of floating debris that was not removed. Adjustment should be performed to remove debris from images in the future.

To examine the utility of our method with other cell lines and culture conditions, an hiPSC line, Tic (JCRB1331)^26^,^27^,^28^, was cultured on vitronectin in a chemically defined serum-free culture medium, TeSR^TM^-E8^TM^, without feeders in a cell culture observation system to obtain colony morphology images. Cluster analysis was performed based on colony morphological parameters. Six major clusters (Major Tic 1–6) were identified for Tic cell culture (Supplementary Fig. S8). Compared to other clusters in the colony database, ‘Major Tic 3’ and ‘Major Tic 4’ were similar to cluster-B. ‘Major Tic 6’ was similar to cluster-C. Thus, the colonies constituting cluster-B and cluster-C represent the most common hPSC morphologies.

### Immunocytochemical staining of colonies

All plates imaged to construct the colony database library were individually fixed with paraformaldehyde after image acquisition and individually stained with various antibodies. Figure 1B shows a representative cluster-A colony that lost an undifferentiated stem cell marker, OCT-3/4 expression in the peripheral region (Fig. 1Ba,b), whereas a representative cluster-B colony expressed OCT-3/4 throughout the colony (Fig. 1Bc,d). Another representative cluster-A colony exhibited an early differentiated marker, VIMENTIN expression in the peripheral region (Fig. 1Be,f), whereas another cluster-B colony exhibited low levels of VIMENTIN expression (Fig. 1Bg,h). Based on these results, the cluster-A morphological category is associated with unstable or undifferentiated colony states.

To further confirm the marker expression rates in characteristic clusters, 201B7 and 201B7-1A cells cultured over 3 passages were evaluated (independent of the colony database or gene expression profile analysis, Supplementary Table S8). Representative colony morphologies and OCT-3/4 and VIMENTIN staining are shown in Supplementary Fig. S8. Immunopositive ratios were calculated by quantifying the marker stained area per single colony (Table 1). In the case of 201B7-1A, both cluster-A and cluster-J colonies, which were small, demonstrated low OCT-3/4 and high VIMENTIN expression ratios. Cluster-A colonies of 201B7 cells exhibited a low OCT-3/4 expression ratio. The small colonies of cluster-I demonstrated both low OCT-3/4 and high VIMENTIN expressions ratios.

### Global gene expression profiles of colonies analysed by morphological parameters

To examine the relationship between gene expression profiles and classification based on our morphological parameters, 201B7 and 201B7-1A cells were freshly cultured (independent of the colony database or marker expression rates) and re-analysed according to morphological parameters after colony database construction as described above (a schema of the experimental flow is shown in Supplementary Fig. S9). Referencing the colony database, images of live 201B7 and 201B7-1A colonies were classified into clusters. Thirty-two representative colonies from major cluster-A, cluster-B and cluster-D and minor cluster-I and cluster-J were selected to be individually and mechanically isolated from the culture vessel (the colony images are shown in Supplementary Fig. S10); 16 of 201B7 colonies (cluster-A = 3, cluster-B = 3, cluster-D = 5, cluster-I = 4, cluster-J = 1) and 16 of 201B7-1A colonies (cluster-A = 6, cluster-B = 3, cluster-D = 4, cluster-I = 2, cluster-J = 1) were designated with the colony IDs described above (Supplementary Table S9). RNA was individually extracted from each colony and analysed using human gene expression microarrays.

**Figure 2:**
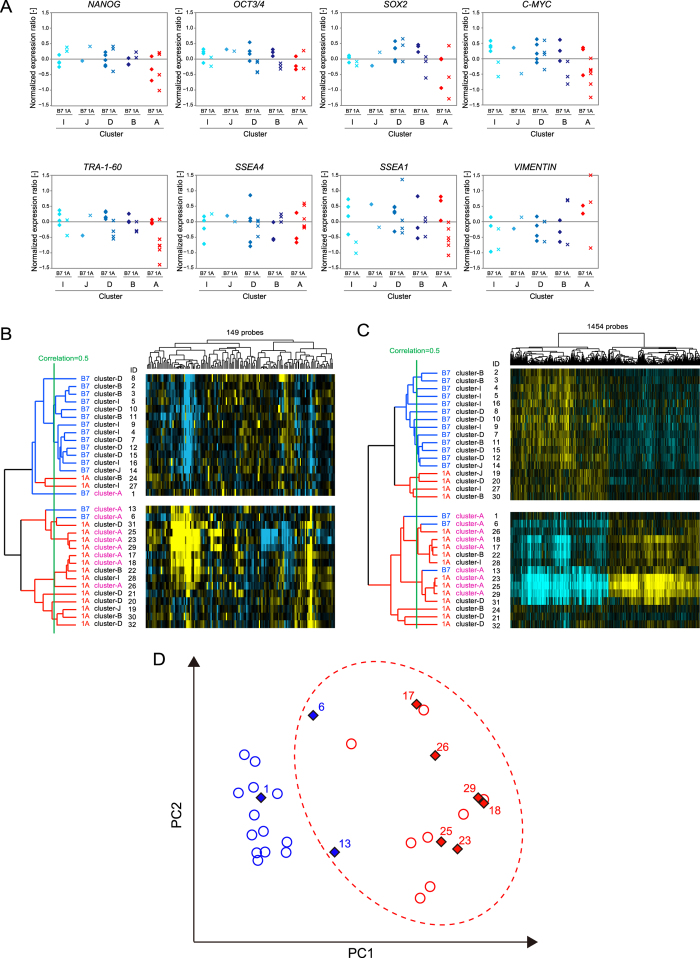
Gene expression profiles of single hiPSC colonies classified as cluster-A, cluster-B, cluster-D, cluster-I and cluster-J 201B7 and 201B7-1A hPSC colonies (32 colonies), classified as cluster-A, cluster-B, cluster-D, cluster-I and cluster-J, were individually picked up from the culture vessel. RNA extracted from these colonies was used to perform global gene microarray analysis. Gene expression profiles are normalised values, as described in the Methods section. (**A**) Comparisons between 201B7 and the aberrant subclone 201B7-1A classified as cluster-A, cluster-B, cluster-D, cluster-I and cluster-J employing representative stem cell markers. (**B**) Hierarchical clustering of the colonies based on 149 probes of stem cell-related markers proposed by the International Stem Cell Initiative^11^. (**C**) Hierarchical clustering of the colonies based on 1,454 probes expressed at significantly higher levels in colonies classified in cluster-A vs. those in cluster-B, cluster-D, cluster-I and cluster-J. (**D**) PCA of colonies based on 29,445 global probes. Blue-filled diamonds: cluster-A colonies in 201B7; red-filled diamonds: cluster-A colonies in 201B7-1A; blue open circles: 201B7 colonies in other clusters; and red open circles: 201B7-1A colonies in other clusters. The numbers on the filled diamonds indicate colony ID numbers in 201B7. The red dotted area indicates biological similarities between colonies, reflecting the expression profile of 29,445 global probes.

First, the expression of the representative undifferentiated stem cell markers *NANOG, OCT-3/4, SOX2, C-MYC, TRA-1-60* and *SSEA4* and the early differentiated cell markers *SSEA1* and *VIMENTIN* was determined from global gene profiles and compared between clusters (Fig. 2A). Among both the 201B7 and 201B7-1A cluster-A colonies, there were large variations in the gene expression levels of *NANOG*, *SOX2* and *VIMENTIN*. Greater variation was observed for 201B7-1A clusters compared with 201B7 clusters. Large variations in *OCT-3/4*, *C-MYC* and *TRA-1-60* expression were observed in cluster-A 201B7-1A colonies. Conversely, the gene expression levels of *NANOG*, *OCT-3/4*, *SOX2* and *TRA-1-60* were comparable between cluster-I, cluster-J, cluster-D and cluster-B for both 201B7 and 201B7-1A. These results reveal greater variations in the gene expression levels of a proportion of undifferentiated or differentiated markers among cluster-A colonies, indicating that cells in cluster-A colonies are unstable and in a dysregulated undifferentiated state.

Next, to understand the stem cell characteristics of the classified colonies, the gene expression of 149 probes proposed by the International Stem Cell Initiative^11^ as undifferentiated or early differentiated hPSC markers was extracted from global gene expression profiles. According to a simple hierarchical cluster analysis, cluster-A colonies of both 201B7 and 201B7-1A clustered closely together (Fig. 2B). Moreover, two 201B7 colonies (no. 6 and no. 13) clustered together with 201B7-1A cluster-A colonies. Although the cluster-A colony (no. 1) of 201B7 was associated with the 201B7 branch, its expression profile demonstrated the greatest divergence among the 201B7 colonies. The expression levels of fibronectin 1 (*FN1*), alpha-fetoprotein (*AFP*), interleukin 6 signal transducer/gp130/oncostatin M receptor (*IL6ST*) and leukaemia inhibitory factor receptor alpha (*LIFR*) were higher (>4-fold) in cluster-A colonies than in other major clusters (genes in blue in Supplementary Table S10). Thus, both 201B7 and 201B7-1A cluster-A colonies exhibit an unstable undifferentiated state.

To explore the characteristic genes in cluster-A colonies, gene expression profiles of individual colonies were compared among clusters. When cluster-B/cluster-D/cluster-I/cluster-J colonies were individually compared with the remaining cluster colonies (1 cluster vs. others, including cluster-A), no statistically significant differences in gene expression levels were observed. Conversely, when cluster-A colonies were compared with the remaining clusters (1 cluster vs. others, excluding cluster A), the expression levels of 1,454 probes differed significantly, suggesting these genes reflect the individual biological characteristics of cluster-A colonies. When hierarchical clustering was performed based on this list of cluster-A-characteristic genes, all cluster-A colonies of 201B7 (no. 1, 6 and 13) demonstrated a higher correlation with cluster-A colonies of 201B7-1A (Fig. 2C). Of the 1,454 identified genes, the expression of 188 genes was lower and that of 399 genes was higher in cluster-A than in other clusters (fold change: >3). Furthermore, 83 genes exhibited a >4-fold change in expression levels (listed in Supplementary Table S10). For example, the expression of myocardial infarction associated transcript (*MIAT*) was lower. Knockdown of *Miat* reportedly alters *Oct-3/4* transcript levels and modulates mESC differentiation^29^. Conversely, the expression levels of genes related to epithelial to mesenchymal transition (EMT), such as forkhead box Q1 (*FOXQ1*)^29^, tenascin C (*TNC*)^30^, S100 calcium-binding protein A14 (*S100A14*)^31^, transforming growth factor beta-induced (*TGFBI*)^32^, chemokine (C-X-C motif) receptor 7 (*CXCR7*)^33^, and chemokine (C-X-C motif) receptor 4 (*CXCR4*)^34^, were higher (EMT-related genes are listed in yellow in Supplementary Table S10). EMT-related genes found on chromosome 12, such as *TGFBI* and frizzled family receptor 4 (*FZD4*)^35^, are listed in Supplementary Table S11. Spontaneous differentiation of hPSC colonies is occasionally observed, as evidenced by VIMENTIN expression at the periphery of hPSC colonies, indicating that EMT occurs in hPSC cultures^36^,^37^. Accordingly, VIMENTIN expression was observed at the periphery of cluster-A colonies (Fig. 1B); thus, the morphology of cluster-A is associated with EMT-related gene expression profiles. Furthermore, the expression of these genes was indicative of the following processes, as identified by gene ontology analysis: ‘developmental process’, ‘locomotion’, ‘multicellular organismal process’, ‘cellular component organization or biogenesis’, ‘biological regulation’, ‘response to stimulus’ and ‘biological adhesion’ (Table 2). Principal component analysis (PCA) of all probes (29,445 probes) demonstrated that the cluster-A colonies in 201B7 (no. 6 and 13) were positioned close to the 201B7-1A colonies (Fig. 2D). Among the 201B7 colonies, 3 were defined as ‘cluster-A colony morphology’. These 201B7 colonies exhibited gene expression profiles similar to those of 201B7-1A colonies. Based on these results, our quantitative morphological evaluation method is able to detect unhealthy colonies prior to definitive diagnosis via gene expression analysis or karyotypic analysis without damaging or wasting cells.

### Colony morphology in cluster-A

An hESC line, H9^1^ (WA09), cultured in our laboratory exhibited genomic instability after prolonged culture and occasionally generated cell populations with aberrant karyotypes (Supplementary Table S3). When aberrant karyotype cell populations appeared, the cells subsequently exhibited a partially differentiated colony morphology, and we experienced difficulty in maintaining an undifferentiated colony morphology. To evaluate the ability of our classification method to recognise cluster-A morphology for the H9 cell population, images of H9 cells cultured on mouse embryonic fibroblasts (MEFs) in KSR-based medium were collected with our cell culture observation system. The colony morphological parameters were first measured prior to cluster analysis, revealing 4 major clusters (Major ES 1–4) among H9 cell colonies (Supplementary Fig. S11). Compared with clusters in the colony database, cluster ‘Major ES1’ was similar to cluster-B, and cluster ‘Major ES3’ was similar to cluster-C. Next, the morphological data for H9 cell colonies were compared with cluster-A data. Referencing the colony database revealed a small number of cluster-A H9 colonies (1.3%, 7 colonies in 523 colonies, Supplementary Table S12). When colony morphologies were manually assessed, colonies classified as cluster-A among H9 cells exhibited irregular morphologies with collapsed edges (Supplementary Fig. S12). Once we clone the aberrant H9 subclone, we intend to implement our quantitative morphological evaluation method to compare this aberrant subclone with normal H9 cells.

## Discussion

Various analytical methods are currently used to evaluate the conditions of undifferentiated, healthy hPSCs. Among them, hPSC colony morphology is a practical and key criterion to assess the quality of hPSCs maintained under laboratory conditions. However, evaluating the morphology of live, intact, non-stained colonies is neither quantitative nor objective. Although various commercially available high-content/high-throughput image acquisition systems demonstrate a certain utility when examining non-stained hPSC colony morphology^10^,^18^,^19^,^20^, the approach described here is unique in that it uses an automated phase-contrast microscopic system to analyse the experimental procedures without arbitrary interference. In the present study, our analysis revealed several new concepts. First, we tuned the auto-image acquisition conditions of the cell culture observation system to facilitate stable and large-scale image data collection. This tuning, which minimises artefacts through image processing, is a crucial component of our non-labelled quantitative morphological evaluation method compared with fluorescent-based cell image analysis. Given that the characteristics and varieties of colony morphologies may vary more widely than can be appreciated during direct observation by human investigators, computational training with selected colony images limits the scope of identifiable cell morphologies. Therefore, all colony varieties in target samples were measured using this tuned auto-image acquisition system to objectively evaluate morphological characteristics. Second, we introduced a clustering concept to reveal ‘representative morphology’ without human bias. In the present study, clustering of data from >300 colonies led to the identification of a ‘major cluster’ based on the combination of multiple morphological parameters and cluster size. The morphology of the major cluster is representative of the corresponding cell line. In the present study, the ‘supervised learning method’ was not used, although this method can effectively ‘train’ computers when certain morphological features are defined by experts with extensive experience or specific marker expression. Given that true morphological features or definitive markers that characterise healthy hPSCs have yet to be identified, it is extremely difficult to assess the undifferentiated state of hPSCs. Compared with the dynamic changes in morphology observed during hiPSC generation, morphological variations in hPSCs during maintenance of the undifferentiated state are sensitive, and hPSC quality is more difficult to define biologically. The measurement of any single marker is unable to provide sufficient data to permit the identification of ‘unhealthy’ colonies. From this perspective, the ‘unsupervised method’, which uses cluster analysis, is an appropriate and objective method to evaluate colony morphology. Third, our morphological evaluation method is applicable to other cell lines. We previously developed growth factor-defined, serum- and feeder-free culture conditions for hESCs^38^ and demonstrated slight variations in colony growth rates and morphology. However, we also analysed the morphology of a different hPSC line, Tic, under different culture conditions. Recently, we reported a method to assess the growth of hPSCs from non-labelled live-cell phase-contrast images using a time-lapse imaging system without damage these cells^25^. Although the equation coefficients to calculate cell numbers based on images differ under different culture conditions, the equation itself remains unchanged. Thus, our evaluation method based on the morphologies of various hPSC lines is applicable under different culture conditions.

To develop a method to evaluate hiPSC colony morphology, we hypothesised that the morphology of each colony exhibits characteristics reflected in individual gene expression profiles. We confirmed our hypothesis by demonstrating that the cluster analysis results for colony morphologies were similarly reproduced by individual gene expression profiles. When gene expression profiles were analysed, 1,454 genes were related to ‘unusual morphology of cluster-A colonies’. Of these, the expression of EMT-related genes was particularly noteworthy. Spontaneous differentiation of normal hPSC colonies is occasionally observed, characterised by VIMENTIN expression at the colony periphery^39^,^40^, whereas 201B7-1A frequently produces colonies with irregularly collapsed colony edges and unclear borders. Thus, EMT frequently occurs in cluster-A colonies, indicating early differentiation attributable to the unstable undifferentiated state of cluster-A colonies. Fibroblast growth factor-2 (FGF-2)-induced phosphoinositide 3-kinase/Akt signalling supports the undifferentiated state of hPSCs^36^. Furthermore, FGF-2-induced protein kinase C initiates EMT in hPSCs, thus highlighting the necessity for a balance between self-renewal and hPSC differentiation to maintain an undifferentiated state^37^. In a previous study, a variant of an hESC line with neoplastic features, including high proliferative capacity and growth factor independence, exhibited morphological differences compared to other hESC lines, including the absence of well-defined colony edges^37^. EMT likely occurs in this variant cell culture because increased cell motility results in the absence of well-defined colony edges. A recent study reported increased cell motility among aberrant hESCs^10^. EMT occurs in cells that detach from the epithelial layer and increases motility during migration to a new location during embryogenesis, tumorigenesis or metastasis *in vivo*^39^. The expression of EMT markers correlates with cell morphology and motility^41^. Thus, EMT is a key process involved in the morphological changes in hESCs/iPSCs.

Based on single-colony gene expression profiling in the present study, 201B7 colonies defined as having ‘cluster-A colony morphology’ demonstrated gene expression profiles similar to 201B7-1A subclone cluster-A colonies. Image analysis of hESC H9 cells utilizing our method demonstrated the presence of colonies with ‘cluster-A colony morphology’. Normal practice in our laboratory is to manually remove partially differentiated colonies with irregular morphology under the microscope prior to passage. If colonies are analysed using the quantitative morphological evaluation method and removed at every passage, good-quality H9 cells can be continuously maintained.

In the present study, we used a cell culture model exhibiting partially differentiated colony morphology. Culture of the 201B7 cell line and its aberrant subclone hiPSC culture provided valuable insights into the biological status of hPSC cultures. If gene expression analysis of different cell lines is expanded to include a wider variety of morphological categories, more specific genes defining individual morphologically detectable colony characteristics will be identified. Accordingly, the diversity of colony morphology should be expanded, and the correlation between colony morphology and gene expression should be evaluated using other hPSC lines and bioimaging informatic analyses^41^,^42^,^43^.

Here, we demonstrated the utility of morphological analysis to evaluate the quality of hPSCs. Using non-labelled images, hPSC quality can be continuously evaluated from the beginning of culture through the final day without wasting or damaging cells. Although various multi-parametric morphological analyses of fluorescently labelled live cell images have been reported, invasive procedures such as labelling or genetic manipulation are required and cannot be used with cells intended for clinical applications. We believe our method possesses another advantage in permitting ‘continuous evaluation’. Through the accumulation of non-labelled images comprising a history of quality transition, future cellular events, such as differentiation propensity, can be predicted. Our non-invasive morphological evaluation method represents a novel tool for hPSC quality assurance. In addition, such detailed quality control will be greatly facilitated by automated technology, including monitoring technology and robotic production technologies. We are currently collaborating with machine manufacturing companies and academic institutions to develop cell systems for automatic expansion as part of the Stem Cell Evaluation Technology Research Centre project.

## Methods

### Cells and cell culture

Two hiPSC lines, 201B7^2^ and 253G1^3^, and their subclones (201B7-1A and 253G1-B1, respectively) were provided by Dr. Shinya Yamanaka of the Center for iPS Cell Research, Kyoto University. The hiPSC line Tic (JCRB1331) was obtained from the JCRB Cell Bank (NIBIOHN, Ibaraki, Osaka, Japan)^26^,^27^,^28^, and the hESC line H9 (WA09)^1^ was obtained from the WISC Bank (WiCell Research Institute, Madison, WI, USA). Experiments using hESCs were performed following the guidelines for the utilization of hESCs established by the Ministry of Education, Culture, Sports, Science and Technology, Japan, with the approval of each institutional research ethics committee. Detailed information on the cell lines used in the present study is provided in Supplementary Tables S1–3. For 201B7 and 201B7-1A, detailed biological characterization is also provided in Supplementary Fig. S1. For teratoma formation assay in Fig. S1C, iPSCs suspended in DMEM supplemented with ROCK inhibitor were injected into the rear leg muscle or thigh muscle of SCID (C.B-17/lcr-scid/scidJcl) mice (CLEA Japan, Tokyo, Japan) (detail described in the Supplementary Methods). All animal experiments were conducted in accordance with the institutional and Japanese guidelines for animal experiments, genetic recombination experiments, human iPSC experiments, and hESC experiments after approval by the committee meetings for the animal experiments and the safety for genetic recombination experiments, and the institutional ethical review board of National Institutes of Biomedical Innovation, Health and Nutrition, Osaka, Japan. Cell lines were routinely maintained as reported previously^25^,^36^,^44^,^45^. The details are described in the Supplementary Methods. Tic cells maintained under conventional culture conditions were transferred to feeder-free culture conditions as described in the Supplementary Methods.

### Immunohistochemical staining

Immunocytochemistry was performed as described previously^36^. Image analysis was performed using CL-Quant (version 3.0 beta, Nikon, Tokyo, Japan). Primary and secondary antibodies used in the present study are listed in Supplementary Table S13.

### Image acquisition

Phase-contrast colony image acquisition was achieved using a BioStation CT culture incubator, microscope and digital imaging system (Nikon) that was adjusted to optimise the auto-focus and cell image-tiling acquisition functions according to the instructions provided (Supplementary Fig. S3). Colony images were obtained from tissue culture-treated 6-well plates (353046, Becton Dickinson, Franklin Lakes, NJ, USA) at 4 × magnification. The centre of each well was optimized to tile 64 ( = 8 × 8) single-shot images (1,000 × 1,000 pixels^2^, 2,000 × 2,000 μm^2^/image) as the field of view for each well. The optimized field of view covered the maximum area in the 6-well plate to obtain phase-contrast colony images without incorporating any effects of the liquid surface meniscus. Images were saved as PNG files. For the acquisition of all images, the interval was set at 8 h, starting 48 h after seeding to avoid the disruption of colony adhesion. To acquire images to construct the colony database, 4 hiPSC clones (201B7, 201B7-1A, 253G1 and 253G1-B1 at 6 plates/cell clone) were prepared and cultured in the BioStation CT for 6 days (total images obtained = 2,304 images, comprising 64 images from 6–12 wells per clone). To acquire images of the colonies selected for single-colony microarray analyses, 6 plates/clone were freshly prepared approximately 6 months after colony database construction with 201B7 and 201B7-1A and cultured in the BioStation CT for 4 days (total images obtained = 1,536 images, 256 images/plate). All raw images (Ph channel) obtained from the BioStation CT and analytical data are available at https://mega.nz/#!3sJXAYLR!67ksyOiGAz2EjI2QSclNfB3z88u2D-SOvendMbuTTXY (For any additional information about the files, please contact the corresponding author).

### Image collection for morphological analysis

The image collection process for morphological analysis is represented in Step 1-1 in Supplementary Fig. S2. Basic colony image measurements were performed using the image analysis software CL-Quant (version 3.0 beta, Nikon). Colony recognition image processing was carried out as described in the manufacturer’s protocol for CL-Quant (Nikon) (a schematic flow is described in Supplementary Figs S13 and S14), utilizing the following steps: (step 1) flattening background (parameter setting: kernel size = 7 pixels and grey value = 90); (step 2) colony recognition via colony texture training with the machine learning algorithm of CL-Quant (choosing the maximum recognition accuracy among 50 training patterns); (step 3) noise reduction (parameter setting: 2,046 pixels, optimized for manual colony counting of 30 images); (step 4) object filling (parameter setting: 30 pixels); (step 5) manual object cleansing (eliminating the labels of colony-like objects that are not actually colonies or are partial images of colonies by confirming them in a phase-contrast image view); and (step 6) size limitation for ‘colony’ selection among objects (>30,000 pixels = 400 μm^2^ diameter). Using measured image-processing parameter settings, 999 colonies were recognised (>30,000 pixels/colony, excluding extremely small and immature colonies), specifically 490 colonies for 201B7, 254 colonies for 201B7-1A, 136 colonies for 253G1 and 119 colonies for 253G1-B1. We then narrowed our colony database to colonies >1.0 mm in diameter because colonies <1.0 mm in diameter were too young to be representative. The selected colony database comprised 303 mature colonies (201B7, 158 colonies; 201B7-1A, 66 colonies; 253G1, 42 colonies and 253G1-B1, 37 colonies) that were not substantially merged with other individual colonies.

### Measurements for morphological analysis

The measurement process for morphological analysis is represented in Step 1–2 in Supplementary Fig. S2. Selected colonies were first measured according to 120 morphological parameters consisting of default software measurement settings and manually added morphological parameters comprising the major morphological parameters found in other software packages such as Image J (Research Services Branch, National Institute of Mental Health, Bethesda, MD, USA) and MetaMorph (Molecular Devices, Sunnyvale, CA, USA). After converting colony morphology to multiple parameters, highly correlated parameters (Pearson’s correlation coefficient: >0.98) and parameters with a high coefficient of variation (CV; >30) were eliminated to reduce the risk of multicolinearity. Highly correlated parameters were represented by a single parameter that describes and interprets cellular morphology. Eight major parameters comprising a total of 27 sub-parameters were selected as essential for further analysis (Supplementary Table S4). Parameter 3 is sensitive for detecting the smoothness of a colony outline, and parameter 6 is sensitive for detecting the fibrous points of a colony outline.

### Clustering for morphological analysis

The clustering process for morphological analysis is represented in Step 1–3 in Supplementary Fig. S2. Image data were further analysed for colony database construction. Using the selected colony morphological data (303 colonies described by 8 major parameters), average linkage hierarchical clustering (uncentred correlation) was carried out using the open source clustering software Cluster 3.0 (University Tokyo, Human Genome Center, Tokyo, Japan). The clustering results were exported to a.csv file and then analysed with an original program written in R for further pruning and member analysis. To prune the hierarchical clustering tree into ‘clusters,’ a ‘test of no correlation’ based on Pearson’s correlation coefficient was used. We ensured that ‘similar colonies’ passed the ‘test of no correlation’ and pruned the clustering tree with a correlation coefficient r >0.380863, which is the correlation coefficient ‘r-value’ of the 27 parameters. This r-value corresponded to the t-value (t = 2.059539) with a significance level of >0.05. Such pruning resulted in ‘clusters’ of colonies. Among the clusters, we designated clusters that consisted of >5% of all colonies (>15 colonies) as ‘major clusters’ and the others as ‘minor clusters.’ After clustering, the colony existence ratio was compared between 201B7 and 201B7-1A to identify cluster characteristics that defined a ‘201B7-1A clone’, which typically has a ‘disordered morphology.’ The clusters were then ordered according to the characteristics related to 201B7, major-ness and average colony size (Supplementary Table S5). Only cluster-A demonstrated a characteristic colony existence ratio in which the clustered colony ratio was higher for 201B7-1A than for 201B7.

### Image data analysis for colony selection for single-colony microarray

The image data analysis process for colony selection for single-colony microarray is represented in Step 2 in Supplementary Fig. S2. After seeding hPSCs for 72 h, 1,536 images (covering 48, 56, 64 and 72 h) acquired from a fresh batch of hiPSC cultures were processed and measured using the above image processing and image analysis scheme. Newly analysed colonies from the 72-h images were then categorised into clusters-A through -T by searching for the colonies in the colony database possessing the highest Pearson’s correlation coefficients using an original program written in C. New live colonies were labelled by returning the corresponding ‘cluster’ from the search results in the colony database. Among these newly labelled live colonies with cluster names, single colonies that were separated from other neighbouring colonies and could be easily picked were manually selected as candidate colonies for microarray analysis. RNA was individually extracted from each colony that was mechanically harvested from the culture vessel.

### Single-colony microarray analysis

The single-colony microarray analysis process is illustrated in Supplementary Fig. S9. The 32 selected colonies from cluster-A, cluster-B and cluster-D (to evaluate major clusters) and cluster-I and cluster-J (to evaluate minor clusters) were labelled with colony ID numbers (listed in Supplementary Table S9). These colonies were marked under the microscope, and neighbouring colonies were eliminated with a cell scraper. Colonies were lysed with Trizol (Life Technologies, Carlsbad, CA, USA) in a colony ring (7-mm diameter), and total RNA was extracted according to the manufacturer’s protocol. Then, small-scale total RNA was amplified using the WT-Ovation Pico RNA Amplification System (3300-12, Nugen Technologies, Inc., CA, USA) and analysed with a BioAnalyzer (Agilent Technologies, Inc., Santa Clara, CA, USA). SurePrint G3 Human GE microarray kits 8 × 60K ver. 2.0 (G4851A, Agilent Technologies, Inc., San Carlos, CA, USA) were used for microarray analysis carried out by DNA Chip Research, Inc., Yokohama, Japan. Microarray data were analysed with GeneSpringGX (Version 12.1) using the standard operating manual provided by the manufacturer. A 75th percentile shift was selected as the sample normalization scheme, and the standard baseline shift was selected for data normalization. Of the 60,000 probes included in the microarray, 29,445 probes had data suitable for analysis. To statistically analyse selected cluster-related genes, a Benjamini–Hochberg *t*-test with a <0.05 significance level was applied. For gene expression clustering, average linkage hierarchical clustering (uncentred correlation) was carried out with Cluster 3.0. Gene ontology analysis was performed according to the manufacturer’s standard protocol. PCA was performed with SPSS (Ver. 18, IBM Corporation, Armonk, NY, USA).

## Additional Information

**How to cite this article**: Kato, R. *et al.* Parametric analysis of colony morphology of non-labelled live human pluripotent stem cells for cell quality control. *Sci. Rep.* **6**, 34009; doi: 10.1038/srep34009 (2016).

## Supplementary Material

Supplementary Information

Supplementary Video S1

Supplementary Video S2

## Figures and Tables

**Table 1:** Marker staining rate in each colony.

Cell	Cluster	Marker staining rate in each colony [%]		Colony number
		OCT-3/4	VIMENTIN	
201B7	A	54.4	0.9	2
	B	89.4	0.7	3
	D	87.0	0.6	2
	I	58.1	2.1	3
	J	77.9	0.8	3
201B7-1A	A	27.1	13.6	3
	B	85.6	0.5	4
	D	81.4	1.2	4
	I	69.5	4.9	3
	J	34.0	14.1	3

**Table 2:** GO analysis of genes expressed in cluster-A.

GO Term	GO accession number	Corrected p-value [−]	Presence of gene in selected probe sets		Presence of gene in all probe sets	
			Count in Selection [genes]	% Count in Selection [%]	Count in Total [genes]	Count in Total [%]
Developmental process	GO:0032502	1.30.E-09	313	32.0	3829	22.1
Locomotion	GO:0040011	8.30.E-06	93	9.5	890	5.1
Multicellular organismal process	GO:0032501 GO:0050874	2.70.E-04	363	37.1	5168	29.8
Cellular component organization or biogenesis	GO:0071840	6.44.E-04	259	26.5	3508	20.2
Biological reglation	GO:0065007	1.18.E-02	580	59.2	9174	52.9
Response to stimulus	GO:0050896 GO:0051869	1.93.E-02	417	42.6	6347	36.6
Biological adhsion	GO:0022610	4.69.E-02	65	6.6	723	4.2
