# Supplementary material for: Parametric analysis of colony morphology of non-labelled live human pluripotent stem cells for cell quality control
Source: Sci Rep. 2016 Sep 26;6:34009. doi: 10.1038/srep34009 (PMC5036041; doi:10.1038/srep34009)
Supplement: Supplementary Information [file srep34009-s1.pdf]

Supplementary Information

Parametric analysis of colony morphology of non-labelled live human pluripotent stem cells for cell quality control

Ryuji Kato[1,2*], Megumi Matsumoto[3], Hiroto Sasaki[3], Risako Joto[1], Mai Okada[1], Yurika Ikeda[1], Kei Kanie[1], Mika Suga[4], Masaki Kinehara[4,5], Kana Yanagihara[4], Yujung Liu[4], Kozue Uchio-Yamada[6], Takayuki Fukuda[4], Hiroaki Kii[2,7], Takayuki Uozumi[2,7], Hiroyuki Honda[3], Yasujiro Kiyota[2,7] and Miho K Furue[2,4*]

[1]Department of Basic Medicinal Sciences, Graduate School of Pharmaceutical Sciences, Nagoya University, Furocho, Chikusa-ku, Nagoya 464-8601, Japan
[2]Stem Cell Evaluation Technology Research Center (SCETRA), Hacho-bori, Chuou-ku, Tokyo 104-0032, Japan
[3]Department of Biotechnology, Graduate School of Engineering, Nagoya University, Furocho, Chikusa-ku, Nagoya 464-8602, Japan
[4]Laboratory of Stem Cell Cultures, National Institutes of Biomedical Innovation, Health and Nutrition, Ibaraki, Osaka 567-0085, Japan
[5] Department of Cellular and Molecular Biology, Basic Life Sciences, Institute of Biomedical & Health Sciences, Hiroshima University, Hiroshima 734-8553, Japan
[6] Laboratory of Animal Models for Human Diseases, National Institutes of Biomedical Innovation, Health and Nutrition, Ibaraki, Osaka 567-0085, Japan
[7]Nikon Corporation, Nagaodaicho, Yokohama, Kanagawa 244-8533, Japan
[*]These authors contributed equally.

Correspondence to:
Miho K Furue (Biological part): Laboratory of Stem Cell Cultures, National Institutes of Biomedical Innovation, Health and Nutrition, Ibaraki, Osaka 567-0085, Japan.
Tel: +81-72-641-9819, Fax: +81-72-641-9812, E-mail address: mkfurue@nibiohn.go.jp

Ryuji Kato (Analysis part): Laboratory of Cell and Molecular Bioengineering, Department of Basic Medicinal Sciences, Graduate School of Pharmaceutical Sciences, Nagoya University, Nagoya 464-8601, Japan.
Tel: +81-52-747-6811, Fax: +81-52-747-6813, E-mail address: kato-r@ps.nagoya-u.ac.jp

Supplementary Methods

hPSC culture

The human Embryonic Stem Cell (hESC) H9 cell line[1], the hiPSC 201B7 cell line[2] and its subclone 201B7-1A, the 253G1 cell line[3] and its subclone 253G1-B1 and the Tic[4-6] cell line were maintained in a KSR-based medium containing DMEM-F12 medium (Life Technologies, Grand Island, NY, USA) supplemented with 20% (v/v) KSR (Life Technologies), 0.1 mM 2-mercaptoethanol (Sigma, St. Louis, MO, USA), MEM non-essential amino acids (Life Technologies) and 4–10 ng/ml recombinant human basic FGF (Katayama Kagaku Kogyo Ltd., Osaka, Japan; 201B7 and 253G1, 4 ng/ml; H9, 5 ng/ml; Tic, 10 ng/ml) as described previously[7,8] on mouse feeder cells (SNL for 201B7 cells, Cell Biolabs, Inc., San Diego, CA, USA; primary mouse fibroblast-P3 CF-1 cells for H9, 201B7 and 253G1 cells and primary mouse fibroblast-P3 BALB/c cells for Tic cells, Millipore, MA, USA) in 1 mg/ml gelatin-coated dishes. The cells were passaged with 1 mg/ml dispase (Roche Applied Science, Penzberg, Upper Bavaria, Germany) and split at a 1:5–1:8 ratio every 6 days. For feeder-free cultures, Tic cells were transferred in TeSR-E8[9] (Stemcell Technologies, Vancouver, BC, Canada) on vitronectin (Stemcell Technologies) and mechanically passaged with a disposable stem cell passaging tool (EZPassage, Life Technologies) as described previously[10]. The cells were split at a 1:5–1:8 ratio every 5 days.

Karyotype analysis

Karyotype analysis was performed as described previously[7,11]. In brief, to obtain a larger number of cells in metaphase, log-phase hiPSCs (3 or 4 days after subculture) were treated with Metaphase Arresting Solution (General Genetic Solutions Ltd., Cheshire, UK) and 0.02–0.04 μg/ml podophyllotoxin (Sigma-Aldrich, St. Louis, MO, USA) for 90–120 min before chromosome preparation. The number of chromosomes was counted in 20 metaphase cells using a Nikon ECLIPSE Ni microscope and NIS Elements Br Software (Nikon, Tokyo, Japan). mFISH staining was performed using a 24XCyte Human Multicolor FISH Probe kit (MetaSystems GmbH Altlussheim, Germany) and analysed with a Zeiss Axio Imager up-light microscope and the Isis FISH Imaging System (Carl Zeiss, AG., Oberkochen, Germany)

Flow cytometry

To test for antigen expression in the hiPSCs, flow cytometry was performed as described previously[7,12]. TRA-1-60, TRA-2-54, TRA-1-81, CD90, SSEA3 and SSEA4 expression was evaluated in each hiPSC line. A FACSCanto flow cytometer (Becton, Dickinson and Company, Franklin Lakes, NJ, USA) was used to acquire data. The FlowJo software (http://www.flowjo.com/) was used for data analyses.

In vitro cell differentiation

In vitro differentiation was induced by the formation of embryoid bodies, as described previously[7,12]. In brief, floating embryoid bodies were maintained in DMEM with 10% FCS for >14 days. Total RNA extracted from cultured cells using an RNeasy Mini kit (Qiagen, Valencia, CA, USA) was treated with DNase I to remove any genomic contamination and reverse-transcribed using a SuperScript VILO cDNA synthesis kit (Invitrogen) according to the manufacturer's instructions. An RT2 Profiler™ PCR Array or TaqMan low density human stem cell pluripotency card PCR array (Applied Biosystems, Foster City, CA) was implemented as described previously[7,12,13]. The expression levels were normalised against the housekeeping gene ^{2} -actin.

Teratoma formation

In vivo differentiation was induced by the injection of hPSCs, as described previously[7,12]. In brief, cells suspended in DMEM supplemented with ROCK inhibitor were injected into the rear leg muscle or thigh muscle of SCID (C.B-17/lcr-scid/scidJcl) mice (CLEA Japan, Tokyo, Japan). Nine weeks after injection, paraffin-embedded sections of induced tumours were stained with hematoxylin and eosin.

Supplementary Table S1. Detailed information for the cells used in the present study

Cell line name	201B7[2]		253G1[3]		Tic[4-6]	H9[1]
Clone name	201B7-1A		253G1-B1			
Generator	Shinya Yamanaka				Akio Umezawa	James Thomson
Origin tissue	Human dermal skin fibroblasts				Human foetus lung fibroblast, MRC-5[14]	Human embryo
Distributor	CiRA, Kyoto University, Kyoto, Japan				Japanese Collection of Research Bioresources (JCRB), Osaka, Japan	WISC Bank, WI, USA
Catalogue No.					JCRB 1331	(WA09)<br>H9-DL-7)
Passage number of distributed cells*	P15	P135	P15	P50	p21+15	p26
Institution of cell culture in this study	National Institutes of Biomedical Innovation, Health and Nutrition (NIBIOHN)					
Culturing history	<6 months	<6 months	<6 months	<6 months	>6 months	>6 months
Cell authentication (STR analysis) after distribution					p21+44 JCRB Cell Bank*	p26+19 JCRB Cell Bank*
Mycoplasma test**	Negative	Negative	Negative	Negative	Negative	Negative
Confirmation of differentiation potential	Teratoma formation***/EB formation	Teratoma formation***/EB formation	EB formation****	EB formation ****	EB formation	EB formation
Karyotype	46,XY	47,XY	46,XY	46,XY*****	46,XX	46,XX

*Cell line authentication using short tandem repeats in cells cultured for >6 months was confirmed by the Japanese Collection of Research Bioresources (JCRB) Cell Bank, National Institutes of Biomedical Innovation, Health and Nutrition (NIBIHON) (Osaka, Japan). 'P' numbers designate passage numbers and '+' designates defrosting.

**Following confirmation of mycoplasma contamination by NIBIOHN or a genetic testing method (PharmaBio Corp. Nagoya, Japan http://www.pharmabio.co.jp/testing-service/virus-bacteria-testing/), mycoplasma contamination was routinely checked every 3 months using a MycoAlert™ mycoplasma

detection kit (Lonza Group Ltd. Basel, Switzerland).
***The differentiation potential of 201B7 cells was confirmed previously via the generation of hiPSCs[2].
****The differentiation potential of 253G1 cells was confirmed previously via the generation of hiPSCs[3].
*****The karyotype of 253G1-B1 cells was 47,XY (Chromosome 46:Chromosome 47 = 43:7) at passage 5 (information provided by CiRA, Kyoto University) but was found to be 46,XY (Chromosome 46 = 50) at passage 42 (information provided by CiRA, Kyoto University).

Supplementary Table S2. *In vitro* differentiation abilities of the cells used in this study

The *in vitro* differentiation abilities of 201B7, 201B7-1A, 2531G1, 253G1-B1, H9 and Tic cells were determined via embryoid body (EB) formation. PCR arrays were performed, and expression levels were normalized against the housekeeping gene ^{2} -actin.

Colors correlate to the fold change in expression of the indicated gene relative to the undifferentiated reference set.

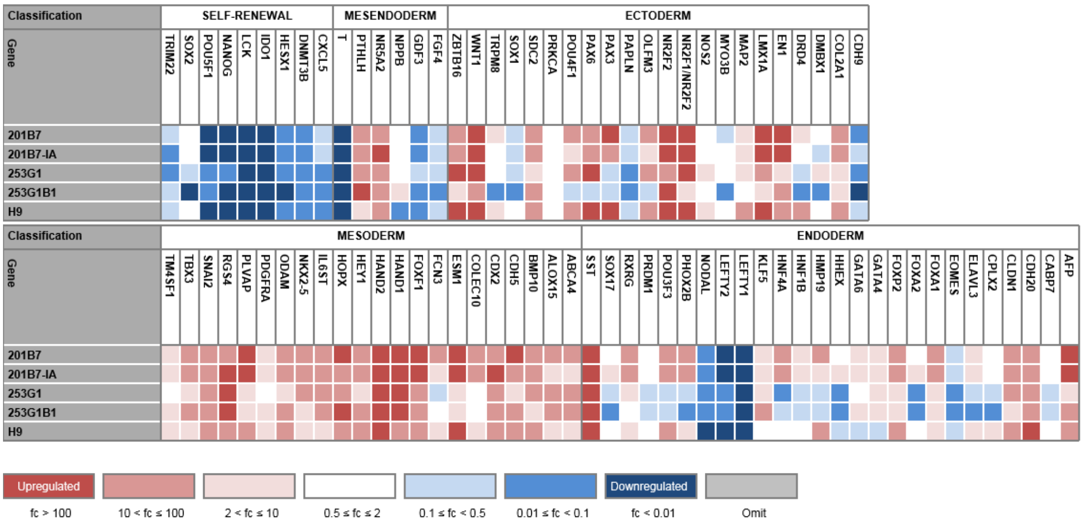

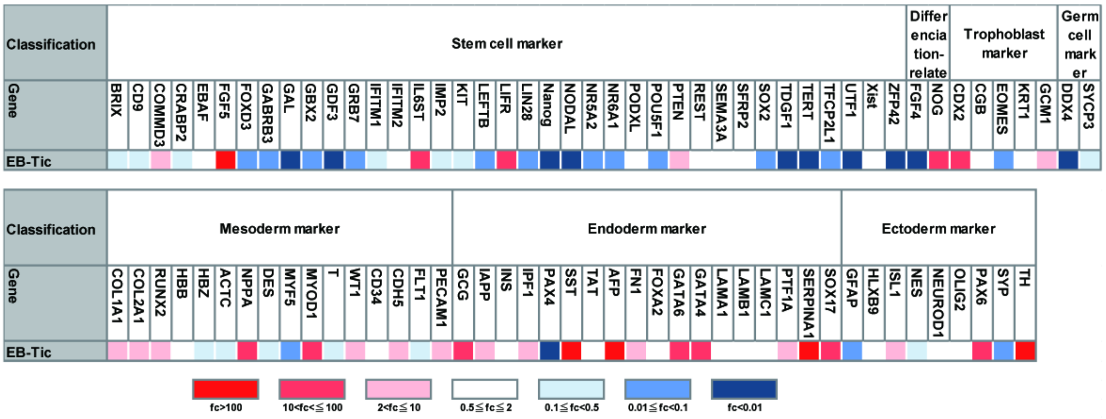

Supplementary Table S3. Culture history information (chromosome numbers counted by standard Giemsa staining)

Number of chromosomes	41	42	43	44	45	46	47	48	49	50
Cell line/passage number	Observed number of metaphases									
201B7, P-21	0	0	0	1	3	46	0	0	0	0
201B7, P-25	0	0	0	0	1	14	0	0	0	0
201B7, P-43	0	0	0	0	0	20	0	0	0	0
201B7-1A, P140	0	0	0	0	0	1	17	2	0	0
201B7-1A, P164	0	0	0	0	0	3	22	0	0	0
253G1, P-24	0	0	0	1	1	27	0	0	0	0
253G1-B1, P-5*	0	0	0	0	0	43	7	0	0	0
253G1-B1,P-42*	0	0	0	0	0	50	0	0	0	0
253G1-B1, P-56	0	0	0	0	0	29	1	0	0	0
H9, p26+42**	1	0	0	0	1	23	1	0	0	0
H9, p26+22**	1	0	0	0	1	16	13	0	0	0
H9, P26+38**	0	0	1	1	1	27	1	0	0	0
H9, p26+16**	0	0	0	0	0	19	0	0	0	0
Tic, p21+51	0	0	0	0	1	19	0	0	0	0
Tic, p21+64	0	0	0	0	0	20	0	0	0	0
Tic, p21+82	0	0	0	1	0	19	0	0	0	0

*Information kindly provided by CiRA, Kyoto University

**H9 cells detected as having aberrant chromosome numbers were discarded (P26+42, P26+22). Then, frozen cell stocks (P26+7) were defrosted again. H9 cells at P26+38 and P26+16 were cultured separately.

Supplementary Table S4. Morphological parameters for colony measurements

	Parameter name	Description	Example
1	Area	Number of pixels	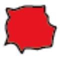
2	Rod-Like Width	Width of most closely surrounding rod-like contour	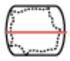
3	Shape Factor	$\frac{4\pi \times \text{Area}}{(\text{Perimeter})^{2}}$	
4	Equivalent Radius	The radius of the circle that would be the same area as the object	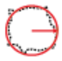
5	Inner Radius	Distance from the centroid to the nearest point along the object edge	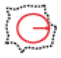
6	Compactness	$\frac{(\text{Perimeter})^{2}}{\text{Area}}$	
7	Perimeter	Perimeter of the object connecting the midpoints of border pixels	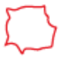
8	Fourier Descriptors: Fourier Descriptors 0<br>Fourier Descriptors 1<br>Fourier Descriptors 2<br>Fourier Descriptors 3<br>Fourier Descriptors 4<br>Fourier Descriptors 5<br>Fourier Descriptors 6<br>Fourier Descriptors 7<br>Fourier Descriptors 8<br>Fourier Descriptors 9<br>Fourier Descriptors 10<br>Fourier Descriptors 11<br>Fourier Descriptors 12<br>Fourier Descriptors 13<br>Fourier Descriptors 14<br>Fourier Descriptors 15<br>Fourier Descriptors 16<br>Fourier Descriptors 17<br>Fourier Descriptors 18<br>Fourier Descriptors 19	Boundary of the object is converted into one-dimensional signal, and then transformed by Fourier transformation. This formation resultsS in 20 frequency components.	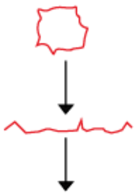 Fourier transformation

Supplementary Table S5. Clustering results for the categorization of morphologically similar iPSC colonies

Cluster Name	Number of colonies assigned to the cluster	Numbers of colonies (%)				Actual size of colonies in the cluster (mm)
		201B7	201B7-1A	253G1	253G1-B1	Maximum
Major Clusters						
A	61	**21 (13.3)**	**18 (27.3)**	**2 (4.8)**	**15 (40.5)**	1.92
B	86	54 (34.2)	15 (22.7)	9 (21.4)	6 (16.2)	1.34
C	36	18 (11.4)	8 (12.1)	0 (0.0)	6 (16.2)	3.39
D	58	20 (12.7)	7 (10.6)	22 (52.4)	0 (0.0)	1.33
E	39	28 (17.7)	6 (9.1)	1 (2.4)	3 (8.1)	2.12
Minor Clusters						
F	25	0 (0)	4 (6.1)	1 (2.4)	1 (2.7)	1.63
G	23	0 (0.6)	2 (3.0)	2 (4.8)	0 (0.0)	1.41
H	17	1 (0.0)	2 (3.0)	0 (0.0)	0 (0.0)	2.17
I	24	4 (2.5)	1 (1.5)	4 (9.5)	2 (5.4)	1.18
J	23	2 (1.3)	1 (1.5)	0 (0.0)	0 (0)	1.33
K	8	1 (0.0)	1 (1.5)	0 (0.0)	1 (2.7)	1.86
L	15	1 (0.6)	1 (1.5)	0 (0.0)	0 (0.0)	2.54
M	12	2 (1.3)	0 (0.0)	0 (0.0)	0 (0.0)	1.33
N	4	1 (0.6)	0 (0.0)	0 (0.0)	0 (0.0)	1.53
O	12	1 (0.6)	0 (0.0)	1 (2.4)	2 (5.4)	1.71
P	8	1 (0.6)	0 (0.0)	0 (0.0)	0 (0.0)	1.8
Q	15	1 (0.6)	0 (0.0)	0 (0.0)	0 (0.0)	2.01
R	21	1 (0.6)	0 (0.0)	0 (0.0)	0 (0.0)	2.12
S	12	1 (0.6)	0 (0.0)	0 (0.0)	1 (2.7)	2.58
T	14	1 (0.6)	0 (0.0)	0 (0.0)	0 (0.0)	2.41

Supplementary Table S6. Interpretation of the morphologies of colonies categorized as cluster-A

Parameter name		Fourier descriptors (0 - 19)		Shape factor		Area / Perimeter / Equivalent radius / Compactness		Inner radius / Rod like width	
Interpretation		Edge frequency at contour		Roundness		Size/volume		Elliptic rate	
		Many edges (weak - strong)	Few edges (weak - strong)	Irregular	Round	Not grown	Well grown	Elliptic	Fat
		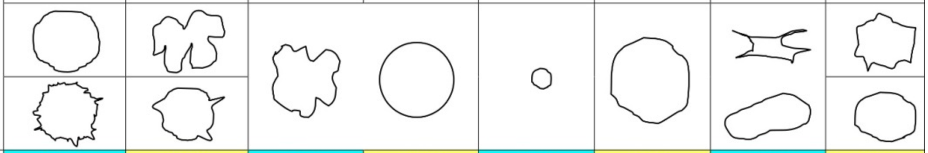							
Parameter value (color in heatmap)		Low (Blue)	High (Yellow)	Low (Blue)	High (Yellow)	Low (Blue)	High (Yellow)	Low (Blue)	High (Yellow)
Cluster name	Cluster-A	○		◎		Variation existing		○	
	Cluster-B	○			○	○		○	
	Cluster-C	○		○			◎		○
	Cluster-D		○		○	○			○
	Cluster-E	○		Variation existing		Variation existing			◎

Supplementary Table S7. Classification results and comparison of morphological parameter values between culture plates rotated 0° and 180°

	Colony number	Colony 1		Colony 2		Colony 3		Colony 4		Colony 5		Colony 6		Colony 7		Colony 8		Colony 9		Colony 10		Colony 11		Colony 12		Colony 13		Colony 14		Colony 15	
	Rotation of plate	Original	180 degrees rotated	Original	180 degrees rotated	Original	180 degrees rotated	Original	180 degrees rotated	Original	180 degrees rotated	Original	180 degrees rotated	Original	180 degrees rotated	Original	180 degrees rotated	Original	180 degrees rotated	Original	180 degrees rotated	Original	180 degrees rotated	Original	180 degrees rotated	Original	180 degrees rotated	Original	180 degrees rotated	Original	180 degrees rotated
	Colony ID in new data	Object 6	Object 29	Object 28	Object 10	Object 15	Object 21	Object 30	Object 9	Object 21	Object 14	Object 9	Object 30	Object 12	Object 26	Object 16	Object 20	Object 33	Object 7	Object 29	Object 8	Object 27	Object 12	Object 32	Object 6	Object 14	Object 23	Object 18	Object 16	Object 24	Object 11
	Predicted cluster number	A	A	A	A	A	A	B	B	B	B	B	B	B	B	B	B	B	B	B	B	B	B	B	B	B	B	B	B	B	A
	Prediction match/mistake	Match		Match		Match		Match		Match		Match		Match		Match		Match		Match		Match		Match		Match		Match		**Mistake**	
	Most similar colony ID in colony database	IA_293_micro_14-2_Crop_1_5	IA_221_micro_14-5_Crop_1_27	IA_221_micro_14-5_Crop_1_27	IA_221_micro_14-5_Crop_1_27	IA_293_micro_14-2_Crop_1_5	IA_293_micro_14-2_Crop_1_5	201B7_163_micro_13-4_Crop_1_5	201B7_163_micro_13-4_Crop_1_5	201B7_273_micro_13-3_Crop_1_39	201B7_273_micro_13-3_Crop_1_39	201B7_273_micro_13-3_Crop_1_39	201B7_273_micro_13-3_Crop_1_39	G1B1_131_1206B1_1_3well_Crop_1_28	G1B1_131_1206B1_1_3well_Crop_1_28	G1B1_131_1206B1_1_3well_Crop_1_28	201B7_161_micro_13-4_Crop_1_37	201B7_193_micro_13-6_Crop_1_30	G1B1_131_1206B1_1_3well_Crop_1_28	201B7_273_micro_13-3_Crop_1_39	201B7_161_micro_13-4_Crop_1_37	201B7_273_micro_13-3_Crop_1_39	G1B1_131_1206B1_1_3well_Crop_1_28	201B7_273_micro_13-3_Crop_1_39	201B7_163_micro_13-4_Crop_1_5	201B7_163_micro_13-4_Crop_1_5	G1B1_131_1206B1_1_3well_Crop_1_28	G1B1_131_1206B1_1_3well_Crop_1_28	G1B1_114_1206B1_1_1well_Crop_1_5	G1B1_131_1206B1_1_3well_Crop_1_28	IA_221_micro_14-5_Crop_1_27
	Correlation coefficient (Correlation to the most imilar colony in colony database)	0.896	0.947	0.966	0.963	0.913	0.917	0.899	0.894	0.877	0.913	0.890	0.900	0.916	0.902	0.874	0.876	0.911	0.892	0.884	0.875	0.883	0.912	0.847	0.869	0.874	0.896	0.918	0.870	0.912	0.937
Parameter name	Area	94029	62981	45569	56724	44877	41730	31498	33449	34281	34871	36144	37268	49185	50245	34136	34281	35744	35876	30944	30886	42075	41248	45675	44265	43920	43537	49290	58571	43553	49426
	RodLikeWidth	62	32	29	35	43	37	38	42	51	61	61	65	48	57	48	50	62	44	55	49	66	48	68	61	57	47	38	82	40	37
	ShapeFactor	0.187	0.066	0.074	0.070	0.168	0.135	0.205	0.255	0.378	0.465	0.408	0.458	0.196	0.254	0.303	0.339	0.461	0.218	0.387	0.347	0.405	0.209	0.450	0.326	0.313	0.221	0.127	0.451	0.143	0.098
	EquivalentRadius	173	142	120	134	120	115	100	103	104	105	107	109	125	126	104	104	107	107	99	99	116	115	121	119	118	118	125	137	118	125
	InnerRadius	16	29	59	28	0	0	26	31	12	2	25	26	33	42	34	23	41	73	20	18	25	21	66	63	7	2	56	94	60	64
	Compactness	67.4	189.8	168.9	178.9	74.8	93.1	61.4	49.2	33.3	27.0	30.8	27.4	64.0	49.6	41.5	37.1	27.3	57.7	32.4	36.2	31.0	60.2	27.9	38.5	40.1	56.8	99.1	27.9	87.9	128.2
	Perimeter	2517	3457	2774	3186	1832	1971	1391	1283	1068	971	1055	1011	1774	1578	1190	1128	987	1439	1002	1058	1142	1576	1129	1306	1327	1572	2210	1278	1957	2517
	FourierDescriptors0	1.522	3.427	1.491	1.878	4.769	3.510	4.852	8.161	2.637	1.971	4.171	3.917	2.496	2.531	3.228	2.950	6.915	1.146	3.508	3.792	1.875	1.621	11.535	10.850	1.957	1.762	2.096	2.592	3.979	5.549
	FourierDescriptors1	3.014	3.970	1.525	1.471	1.259	2.083	0.721	1.123	1.099	0.871	2.735	2.320	1.632	1.747	1.194	1.146	1.645	5.418	0.818	0.817	1.690	1.388	2.418	2.513	1.850	1.660	4.284	6.491	1.862	0.291
	FourierDescriptors2	0.915	4.050	1.187	1.001	2.108	1.338	1.777	2.482	0.379	0.223	1.529	0.934	0.602	0.648	0.938	0.801	1.145	0.483	1.023	1.108	0.513	0.431	5.392	5.315	1.241	0.986	1.742	2.678	0.839	1.553
	FourierDescriptors3	0.892	1.447	0.848	0.046	1.344	0.962	1.583	1.775	0.718	0.654	0.828	0.722	0.493	0.495	1.223	1.038	1.251	1.401	0.730	0.561	0.497	0.472	2.189	2.003	0.764	0.632	0.681	1.386	2.458	2.428
	FourierDescriptors4	1.058	0.732	1.153	0.917	0.724	0.621	0.929	2.085	0.486	0.603	0.210	0.498	1.226	1.143	0.358	0.371	0.503	1.322	0.452	0.376	0.302	0.292	3.898	4.171	0.247	0.228	1.794	2.999	0.281	1.786
	FourierDescriptors5	0.431	1.710	0.323	0.676	0.721	1.095	0.885	0.642	0.342	0.100	0.567	1.002	0.766	0.711	0.286	0.165	0.735	0.574	0.251	0.330	0.500	0.415	0.591	0.418	0.765	0.686	1.183	1.729	0.737	1.040
	FourierDescriptors6	0.236	0.638	0.435	0.161	0.718	0.262	0.426	1.125	0.209	0.184	0.973	0.594	0.186	0.262	0.554	0.456	0.749	0.704	0.271	0.401	0.637	0.510	1.360	1.038	0.541	0.543	0.786	1.437	0.527	1.658
	FourierDescriptors7	0.494	0.423	0.867	0.470	0.365	0.265	0.940	0.853	0.281	0.306	0.693	0.554	0.183	0.057	0.472	0.376	0.245	0.505	0.044	0.173	0.325	0.316	1.008	1.066	0.328	0.200	0.808	0.546	0.337	0.676
	FourierDescriptors8	0.076	0.214	0.308	0.413	0.324	0.447	0.416	0.198	0.390	0.235	0.352	0.197	0.167	0.268	0.268	0.244	0.261	0.322	0.074	0.159	0.178	0.162	0.582	0.572	0.066	0.068	0.743	1.146	0.482	0.549
	FourierDescriptors9	0.456	1.096	0.101	0.167	0.286	0.246	0.379	0.475	0.052	0.101	0.136	0.362	0.180	0.248	0.540	0.446	0.505	0.355	0.094	0.187	0.307	0.254	0.738	0.792	0.201	0.131	0.995	0.727	0.145	0.558
	FourierDescriptors10	0.278	0.088	0.160	0.398	0.187	0.497	0.082	0.842	0.270	0.176	0.370	0.264	0.404	0.340	0.386	0.358	0.109	0.416	0.131	0.250	0.351	0.143	0.271	0.477	0.101	0.109	0.733	0.700	0.452	0.425
	FourierDescriptors11	0.241	0.694	0.138	0.153	0.447	0.441	0.510	0.601	0.200	0.210	0.198	0.434	0.363	0.277	0.276	0.199	0.546	0.333	0.057	0.080	0.131	0.097	0.679	0.686	0.111	0.158	0.323	0.339	0.230	0.350
	FourierDescriptors12	0.239	0.529	0.115	0.127	0.323	0.218	0.212	0.261	0.129	0.105	0.328	0.163	0.404	0.463	0.280	0.271	0.422	0.336	0.142	0.106	0.068	0.074	0.743	0.510	0.222	0.214	0.410	0.160	0.444	0.110
	FourierDescriptors13	0.488	0.684	0.272	0.179	0.036	0.152	0.305	0.568	0.228	0.025	0.057	0.155	0.287	0.201	0.197	0.224	0.244	0.187	0.144	0.140	0.132	0.142	0.479	0.616	0.317	0.296	0.350	0.429	0.170	0.618
	FourierDescriptors14	0.188	0.718	0.311	0.308	0.220	0.416	0.306	0.239	0.054	0.092	0.326	0.295	0.161	0.139	0.144	0.138	0.288	0.174	0.107	0.012	0.182	0.130	0.509	0.510	0.158	0.104	0.194	0.302	0.196	0.739
	FourierDescriptors15	0.169	0.421	0.034	0.136	0.410	0.013	0.305	0.373	0.178	0.118	0.155	0.332	0.205	0.224	0.078	0.086	0.322	0.185	0.028	0.181	0.056	0.108	0.820	0.579	0.136	0.169	0.433	0.466	0.118	0.352
	FourierDescriptors16	0.435	0.666	0.133	0.140	0.113	0.297	0.218	0.554	0.162	0.065	0.228	0.192	0.262	0.284	0.197	0.234	0.181	0.070	0.060	0.067	0.172	0.097	0.439	0.251	0.111	0.126	0.308	0.452	0.187	0.437
	FourierDescriptors17	0.067	0.636	0.177	0.172	0.216	0.151	0.155	0.455	0.047	0.043	0.078	0.224	0.172	0.257	0.255	0.144	0.071	0.224	0.075	0.061	0.167	0.099	0.640	0.543	0.185	0.219	0.412	0.661	0.181	0.167
	FourierDescriptors18	0.170	0.441	0.195	0.009	0.130	0.087	0.226	0.091	0.122	0.074	0.180	0.064	0.084	0.097	0.139	0.139	0.062	0.161	0.047	0.069	0.117	0.120	0.120	0.187	0.183	0.119	0.042	0.313	0.299	0.129
	FourierDescriptors19	0.286	0.185	0.151	0.203	0.065	0.243	0.028	0.352	0.088	0.090	0.288	0.186	0.195	0.163	0.210	0.148	0.068	0.186	0.030	0.085	0.070	0.067	0.520	0.438	0.072	0.050	0.389	0.764	0.172	0.170

Supplementary Table S8. Detailed information for cells used for marker expression analysis and their colony classification results

	201B7			201B7-1A		
	Exp 1	Exp 2	Exp 3	Exp 1	Exp 2	Exp 3
Passage number of used cells*	15+5+4 +8	15+5+4+9	15+5+4 +10	135+3+4+ 3+5+4+8	135+3+4+ 3+5+4+9	135+3+4+ 3+5+4+10
Length of culture [days]	12	12	10	3	5	9
Medium change	Yes	No***	No***	Yes	Yes	Yes
Cluster A [colonies]	0	33	3	30	19	28
Cluster B [colonies]	ND**	5	67	97	38	125
Cluster D [colonies]	ND**	8	34	38	8	20
Cluster I [colonies]	ND**	7	3	17	9	5
Cluster J [colonies]	ND**	1	3	2	2	2

* '+' designates defrosting.

** Not determined. As there were no cluster-A colonies, colony analysis was not performed.

*** Medium was not changed to create stressful culture conditions. With medium change, cluster-A colonies were not found among cells with the same passage number (201B7).

Supplementary Table S9. List of colonies selected for single colony global gene expression analysis

Colony ID	Cell name	Assigned cluster name
1	201B7	A
2	201B7	B
3	201B7	B
4	201B7	I
5	201B7	I
6	201B7	A
7	201B7	D
8	201B7	D
9	201B7	I
10	201B7	D
11	201B7	B
12	201B7	D
13	201B7	A
14	201B7	J
15	201B7	D
16	201B7	I
17	201B7-1A	A
18	201B7-1A	A
19	201B7-1A	J
20	201B7-1A	D
21	201B7-1A	D
22	201B7-1A	B
23	201B7-1A	A
24	201B7-1A	B
25	201B7-1A	A
26	201B7-1A	A
27	201B7-1A	I
28	201B7-1A	I
29	201B7-1A	A
30	201B7-1A	B
31	201B7-1A	D
32	201B7-1A	D

Supplementary Table S10. Genes demonstrating significantly different expression levels in cluster-A (fold change: >4)

Genes in yellow rows are related to EMT. Each representative reference (Ref) is listed below Supplementary Table S8. Genes in blue are the undifferentiated or early differentiated hPSC markers proposed by the International Stem Cell Initiative[12].

Probe ID	Corrected p-value	Fold change	Gene symbol	Description (Homo sapiens)	Ref.
Highly expressed genes (Cluster-A vs. Other clusters)					
A_23_P112859	0.0053	43.6	CST1	cystatin SN (CST1), mRNA [NM_001898]	1
A_23_P203191	0.0035	28.69	APOA1	apolipoprotein A-I (APOA1), mRNA [NM_000039]	2
A_23_P206760	0	19.85	HP	haptoglobin (HP), transcript variant 1, mRNA [NM_005143]	3
A_32_P164246	0.0073	16.98	FOXQ1	forkhead box Q1 (FOXQ1), mRNA [NM_033260]	4
A_23_P58770	0.0073	15.43	HAND1	heart and neural crest derivatives expressed 1 (HAND1), mRNA [NM_004821]	5
A_33_P3298539	0.0034	14.12	APOA1	apolipoprotein A-I (APOA1), mRNA [NM_000039]	2
A_23_P105923	0.0108	13.93	DIO3	deiodinase, iodothyronine, type III (DIO3), mRNA [NM_001362]	6
A_24_P151032	0.0049	11.94	MYL4	myosin, light chain 4, alkali; atrial, embryonic (MYL4), transcript variant 2, mRNA [NM_002476]	7
A_23_P14302	0.0073	11.31	LINC00341	long intergenic non-protein coding RNA 341 (LINC00341), non-coding RNA [NR_026779]	
A_23_P62752	0.0034	10.79	NPPB	natriuretic peptide B (NPPB), mRNA [NM_002521]	
A_23_P157865	0.0073	10.37	TNC	tenascin C (TNC), mRNA [NM_002160]	4
A_23_P124619	0.0034	9.16	S100A14	S100 calcium binding protein A14 (S100A14), mRNA [NM_020672]	8
A_24_P418408	0.0155	9.13	FAM89A	family with sequence similarity 89, member A (FAM89A), mRNA [NM_198552]	
A_23_P77859	0.0134	8.63	TMEM88	transmembrane protein 88 (TMEM88), mRNA [NM_203411]	9
A_23_P156327	0.0181	8.13	TGFBI	transforming growth factor, beta-induced, 68 kDa (TGFBI), mRNA [NM_000358]	4
A_23_P148088	0.0411	8.07	FGG	fibrinogen gamma chain (FGG), transcript variant gamma-A, mRNA	10

				[NM_000509]	
A_23_P59738	0.0049	7.87	MYL7	myosin, light chain 7, regulatory (MYL7), mRNA [NM_021223]	
A_24_P940115	0.0126	7.48	DLC1	deleted in liver cancer 1 (DLC1), transcript variant 1, mRNA [NM_182643]	11
A_23_P98900	0.0075	7.31	CCDC92	coiled-coil domain containing 92 (CCDC92), mRNA [NM_025140]	
A_23_P52121	0.0051	7.3	PDZK1	PDZ domain containing 1 (PDZK1), transcript variant 1, mRNA [NM_002614]	12
A_23_P127584	0.0219	7.27	NNMT	nicotinamide N-methyltransferase (NNMT), mRNA [NM_006169]	13
A_23_P114883	0.0167	7.2	FMOD	fibromodulin (FMOD), mRNA [NM_002023]	14
A_23_P131676	0.0073	6.97	CXCR7	chemokine (C-X-C motif) receptor 7 (CXCR7), mRNA [NM_020311]	15
A_23_P102000	0.0112	6.84	CXCR4	Homo sapiens chemokine (C-X-C motif) receptor 4 (CXCR4), transcript variant 1, mRNA [NM_001008540]	4
A_33_P3271470	0.0073	6.59	GREB1 L	Homo sapiens growth regulation by oestrogen in breast cancer-like (GREB1 L), mRNA [NM_001142966]	16
A_23_P119040	0.0171	6.43	GREB1 L	Homo sapiens growth regulation by oestrogen in breast cancer-like (GREB1 L), mRNA [NM_001142966]	16
A_33_P3304372	0.0093	6.42	TMEM144	Homo sapiens transmembrane protein 144 (TMEM144), mRNA [NM_018342]	
A_23_P33984	0.0138	6.37	TMEM27	Homo sapiens transmembrane protein 27 (TMEM27), mRNA [NM_020665]	17
A_23_P318860	0.0369	6.3	APLNR	Homo sapiens apelin receptor (APLNR), transcript variant 1, mRNA [NM_005161]	18
A_23_P166823	0.0048	6.3	TNNC1	Homo sapiens troponin C type 1 (slow) (TNNC1), mRNA [NM_003280]	19
A_32_P86763	0.0203	6.12	TGM2	Homo sapiens transglutaminase 2 (C polypeptide, protein-glutamine-gamma-glutamyltransferase) (TGM2), transcript variant 1, mRNA [NM_004613]	4
A_24_P20630	0.0147	6.12	LEF1	Homo sapiens lymphoid enhancer-binding factor 1 (LEF1), transcript variant 1, mRNA [NM_016269]	4
A_23_P45786	0.0163	6.08	COL9A2	Homo sapiens collagen, type IX, alpha 2 (COL9A2), mRNA [NM_001852]	20
A_23_P329798	0.0303	5.78	CER1	Homo sapiens cerberus 1, cysteine knot superfamily, homolog (Xenopus laevis) (CER1), mRNA [NM_005454]	21
A_24_P334130	0.0034	5.76	FN1	Homo sapiens fibronectin 1 (FN1), transcript variant 7, mRNA [NM_054034]	4
A_23_P10206	0.0213	5.76	HAS2	Homo sapiens hyaluronan synthase 2 (HAS2), mRNA [NM_005328]	4

A_24_P943393	0.0054	5.71	AHNAK	Homo sapiens AHNAK nucleoprotein (AHNAK), transcript variant 1, mRNA [NM_001620]	22
A_33_P3421243	0.0309	5.5	AFP	Homo sapiens alpha-fetoprotein (AFP), mRNA [NM_001134]	
A_23_P75283	0.0422	5.42	RBP4	Homo sapiens retinol binding protein 4, plasma (RBP4), mRNA [NM_006744]	
A_33_P3362008	0.0038	5.27	NPPB	Homo sapiens natriuretic peptide B (NPPB), mRNA [NM_002521]	
A_23_P214011	0.0054	5.25	CDH6	Homo sapiens cadherin 6, type 2, K-cadherin (foetal kidney) (CDH6), mRNA [NM_004932]	23
A_23_P66682	0.0137	5.24	HOXB6	Homo sapiens homeobox B6 (HOXB6), mRNA [NM_018952]	
A_23_P110167	0.0073	5.2	MGST2	Homo sapiens microsomal glutathione S-transferase 2 (MGST2), transcript variant 1, mRNA [NM_002413]	
A_33_P3629678	0.0073	5.11	COL5A1	collagen, type V, alpha 1 (COL5A1), mRNA [NM_000093]	24
A_24_P687594	0.0126	5.1	LIX1 L	Lix1 homolog (mouse)-like [Source:HGNC Symbol;Acc:28715] [ENST00000369308]	
A_23_P54649	0.0163	5.01	TRADD	TNFRSF1A-associated via death domain (TRADD), mRNA [NM_003789]	
A_23_P214026	0.0205	4.99	FBN2	fibrillin 2 (FBN2), mRNA [NM_001999]	
A_23_P502470	0.0108	4.92	IL6ST	interleukin 6 signal transducer (gp130, oncostatin M receptor) (IL6ST), transcript variant 1, mRNA [NM_002184]	25
A_23_P89431	0.0108	4.73	CCL2	chemokine (C-C motif) ligand 2 (CCL2), mRNA [NM_002982]	26
A_33_P3287338	0.0073	4.73	IL6ST	interleukin 6 signal transducer (gp130, oncostatin M receptor) (IL6ST), transcript variant 3, mRNA [NM_001190981]	25
A_23_P363778	0.0145	4.71	FRZB	frizzled-related protein (FRZB), mRNA [NM_001463]	27
A_23_P215634	0.039	4.69	IGFBP3	insulin-like growth factor binding protein 3 (IGFBP3), transcript variant 1, mRNA [NM_001013398]	28
A_23_P301521	0.0498	4.67	KIAA1462	KIAA1462 (KIAA1462), mRNA [NM_020848]	
A_23_P48596	0.0054	4.65	RNASE1	ribonuclease, RNase A family, 1 (pancreatic) (RNASE1), transcript variant 3, mRNA [NM_198232]	
A_23_P36825	0.0103	4.63	GPRC5A	G protein-coupled receptor, family C, group 5, member A (GPRC5A), mRNA [NM_003979]	29
A_24_P365515	0.0352	4.61	FOXA2	forkhead box A2 (FOXA2), transcript variant 1, mRNA [NM_021784]	20
A_24_P40721	0.0073	4.59	SPTLC3	serine palmitoyltransferase, long chain base subunit 3 (SPTLC3), mRNA	

				[NM_018327]	
A_23_P383819	0.0233	4.58	TBX3	T-box 3 (TBX3), transcript variant 2, mRNA [NM_016569]	4
A_33_P3233834	0.0054	4.57	IL6ST	interleukin 6 signal transducer (gp130, oncostatin M receptor) (IL6ST), transcript variant 3, mRNA [NM_001190981]	25
A_21_P0010978	0.0073	4.5	NEAT1		
A_23_P58588	0.0073	4.47	SLIT3	slit homolog 3 (Drosophila) (SLIT3), mRNA [NM_003062]	
A_23_P253896	0.0333	4.38	NPNT	nephronectin (NPNT), transcript variant 2, mRNA [NM_001033047]	
A_32_P69368	0.0306	4.3	ID2	inhibitor of DNA binding 2, dominant negative helix-loop-helix protein (ID2), mRNA [NM_002166]	4
A_23_P143143	0.0196	4.29	ID2	inhibitor of DNA binding 2, dominant negative helix-loop-helix protein (ID2), mRNA [NM_002166]	4
A_23_P258136	0.0056	4.28	MXRA5	matrix-remodelling associated 5 (MXRA5), mRNA [NM_015419]	
A_24_P397386	0.0056	4.25	LIFR	leukaemia inhibitory factor receptor alpha (LIFR), transcript variant 2, mRNA [NM_002310]	
A_23_P205177	0.0129	4.25	F10	coagulation factor X (F10), mRNA [NM_000504]	
A_24_P27234	0.0313	4.22	SOX5	SRY (sex determining region Y)-box 5 (SOX5), transcript variant 2, mRNA [NM_152989]	30
A_23_P58082	0.0212	4.21	CCDC80	coiled-coil domain containing 80 (CCDC80), transcript variant 1, mRNA [NM_199511]	
A_23_P83098	0.0306	4.2	ALDH1A1	aldehyde dehydrogenase 1 family, member A1 (ALDH1A1), mRNA [NM_000689]	31
A_24_P268676	0.0087	4.11	BHLHE40	basic helix-loop-helix family, member e40 (BHLHE40), mRNA [NM_003670]	32
A_23_P204847	0.0288	4.11	LCP1	lymphocyte cytosolic protein 1 (L-plastin) (LCP1), mRNA [NM_002298]	
A_33_P3233843	0.0098	4.11	IL6ST	interleukin 6 signal transducer (gp130, oncostatin M receptor) (IL6ST), transcript variant 3, mRNA [NM_001190981]	25
A_24_P282309	0.0073	4.1	MYOF	myoferlin (MYOF), transcript variant 2, mRNA [NM_133337]	33
A_21_P0001704	0.0143	4.09	XLOC_000390	EST375352 MAGE resequenced, MAGH Homo sapiens cDNA, mRNA sequence [AW963279]	
A_23_P73114	0.0124	4.07	PROS1	S (alpha) (PROS1), mRNA [NM_000313]	
A_33_P3226832	0.0034	4.07	F3	coagulation factor III (thromboplastin, tissue factor) (F3), transcript variant 1,	

				mRNA [NM_001993]	
A_24_P69095	0.0255	4.07	ENC1	ectodermal-neural cortex 1 (with BTB-like domain) (ENC1), mRNA [NM_003633]	
A_24_P389916	0.0472	4.03	LRRC32	leucine rich repeat containing 32 (LRRC32), transcript variant 1, mRNA [NM_005512]	
Low-expression genes (Cluster-A vs. Other clusters)					
A_24_P854492	0.0375	4.51	MIAT	myocardial infarction associated transcript (non-protein coding) (MIAT), transcript variant 1, non-coding RNA [NR_003491]	
A_19_P00322900	0.0271	4.35	MIAT	myocardial infarction associated transcript (non-protein coding) (MIAT), transcript variant 1, non-coding RNA [NR_003491]	
A_21_P0012853	0.0416	4.06	XLOC_l2_011798	BROAD Institute lincRNA (XLOC_l2_011798), lincRNA [TCONS_l2_00022559]	
A_23_P401055	0.0458	4.05	SOX2	SRY (sex determining region Y)-box 2 (SOX2), mRNA [NM_003106]	

Supplementary Table S11. Genes on chromosome 12 with significantly different expression levels in cluster-A

Genes in yellow rows are related to EMT. Each representative reference (Ref) is listed below.

Genomic coordinates	Probe ID	Corrected P-value	FC	Gene symbol	Description (Homo sapiens)	Ref.
Highly expressed genes (Cluster-A vs. Other clusters)						
chr12:53291085-53291026	A_24_P151032	0.004874	11.9	MYL4	myosin, light chain 4, alkali; atrial, embryonic (MYL4), transcript variant 2, mRNA [NM_002476]	7
chr12:56553503-56553806	A_23_P156327	0.018084	8.13	TGFBI	transforming growth factor, beta-induced, 68 kDa (TGFBI), mRNA [NM_000358]	4
chr12:8482117-8482176	A_23_P122144	0.03748	3.67	UGT3A1	UDP glycosyltransferase 3 family, polypeptide A1 (UGT3A1), transcript variant 1, mRNA [NM_152404]	
chr12:53343300-53343241	A_32_P4199	0.021454	3.61	RNF152	ring finger protein 152 (RNF152), mRNA [NM_173557]	
chr12:89993004-89992945	A_23_P50919	0.01243	3.38	SERPINE2	serpin peptidase inhibitor, clade E (nexin, plasminogen activator inhibitor type 1), member 2 (SERPINE2), transcript variant 1, mRNA [NM_006216]	34
chr12:105760440-105761274	A_23_P10291	0.009192	2.63	CTSE	cathepsin E (CTSE), transcript variant 1, mRNA [NM_001910]	35
chr12:109705969-109706028	A_23_P64617	0.014647	2.36	FZD4	frizzled family receptor 4 (FZD4), mRNA [NM_012193]	36
chr12:53204825-53207427	A_32_P166693	0.021875	2.32	HEG1	HEG homolog 1 (zebrafish) (HEG1), mRNA [NM_020733]	
chr12:132636912-132636971	A_33_P3361741	0.011941	2.27	DNAJC15	DnaJ (Hsp40) homolog, subfamily C, member 15 (DNAJC15), mRNA [NM_013238]	
chr12:50038227-50038286	A_33_P3293456	0.0289	2.21	GATA4	GATA binding protein 4 (GATA4), mRNA	37

					[NM_002052]	
chr12:123956985-123956926	A_19_P00319854	0.032681	2.2	MGC23284	uncharacterized LOC197187 (MGC23284), transcript variant 2, non-coding RNA [NR_024399]	
chr12:27802948-27803007	A_23_P25964	0.00733	2.18	GALC	galactosylceramidase (GALC), transcript variant 1, mRNA [NM_000153]	38
chr12:106461136-106461077	A_23_P12343	0.041077	2.13	GSTM3	glutathione S-transferase mu 3 (brain) (GSTM3), transcript variant 1, mRNA [NM_000849]	39
chr12:121868139-121868080	A_24_P277934	0.041077	2.07	COL1A2	collagen, type I, alpha 2 (COL1A2), mRNA [NM_000089]	40
chr12:7965262-7965203	A_23_P97990	0.041077	1.92	HTRA1	HtrA serine peptidase 1 (HTRA1), mRNA [NM_002775]	41
chr12:56349072-56348089	A_24_P221575	0.033985	1.72	RUFY3	RUN and FYVE domain containing 3 (RUFY3), transcript variant 1, mRNA [NM_001037442]	42
chr12:9092293-9092352	A_24_P810697	0.04483	1.72	MXRA7	Homo sapiens matrix-remodelling associated 7 (MXRA7), transcript variant 2, mRNA [NM_001008529]	43
chr12:56388026-56388085	A_23_P353574	0.041996	1.72	NEK7	NIMA (never in mitosis gene a)-related kinase 7 (NEK7), mRNA [NM_133494]	
chr12:53494853-53494912	A_33_P3494875	0.032878	1.71	LOC100506748	PREDICTED: Homo sapiens hypothetical LOC100506748 (LOC100506748), miscRNA [XR_109878]	
chr12:56119950-56119667	A_23_P200298	0.033985	1.61	AGL	amylo-alpha-1, 6-glucosidase, 4-alpha-glucanotransferase (AGL), transcript variant 4, mRNA [NM_000028]	
chr12:112247448-112247507	A_24_P416177	0.047275	1.58	ADCY7	adenylate cyclase 7 (ADCY7), mRNA [NM_001114]	44
chr12:118503705-118503646	A_23_P134395	0.01689	1.56	TBL2	transducin (beta)-like 2 (TBL2), mRNA [NM_012453]	
chr12:98848490-98848431	A_24_P391260	0.026525	1.55	PTTG1IP	pituitary tumour-transforming 1 interacting protein (PTTG1IP), mRNA [NM_004339]	45

chr12:9093957-9094016	A_32_P133884	0.047035	1.53	TUSC1	tumour suppressor candidate 1 (TUSC1), mRNA [NM_001004125]	46
chr12:52709761-52709702	A_23_P53152	0.021658	1.51	AKIP1	A kinase (PRKA) interacting protein 1 (AKIP1), transcript variant 1, mRNA [NM_020642]	
chr12:115108899-115108840	A_24_P200162	0.021808	1.44	HIGD1A	HIG1 hypoxia inducible domain family, member 1A (HIGD1A), transcript variant 3, mRNA [NM_014056]	
chr12:53822794-53823295	A_21_P0010982	0.035402	1.44	MALAT1	metastasis associated lung adenocarcinoma transcript 1 (non-protein coding) (MALAT1), non-coding RNA [NR_002819]	47
chr12:15095117-15095058	A_23_P4572	0.047301	1.4	MYL12A	myosin, light chain 12A, regulatory, non-sarcomeric (MYL12A), mRNA [NM_006471]	
chr12:53345577-53345636	A_23_P124024	0.04577	1.33	MED10	mediator complex subunit 10 (MED10), mRNA [NM_032286]	
chr12:57865971-57866030	A_33_P3325023	0.031713	1.27	ERLEC1	endoplasmic reticulum lectin 1 (ERLEC1), transcript variant 1, mRNA [NM_015701]	
chr12:13065707-13065766	A_21_P0013647	0.036366	1.16	LOC96610	BMS1 homolog, ribosome assembly protein (yeast) pseudogene (LOC96610), non-coding RNA [NR_027293]	

Low-expression genes (Cluster-A vs. Other clusters)						
chr12:11091141-11091082	A_23_P430181	0.021329	2.39	ZBTB3	zinc finger and BTB domain containing 3 (ZBTB3), mRNA [NM_024784]	
chr12:8482145-8482204	A_23_P19182	0.039048	2.25	REEP2	receptor accessory protein 2 (REEP2), mRNA [NM_016606]	
chr12:107367533-107367592	A_33_P3230188	0.039431	2.24	LINC00328	melanoma antigen mRNA, complete cds. [AF172850]	
chr12:95228736-95228795	A_23_P136623	0.041005	2.23	ADCY5	adenylate cyclase 5 (ADCY5), transcript variant 1, mRNA [NM_183357]	
chr12:23685353-23685294	A_23_P332326	0.012931	2.22	ARHGEF19	Rho guanine nucleotide exchange factor (GEF) 19	

					(ARHGEF19), mRNA [NM_153213]	
chr12:54789715-54789656	A_33_P3408757	0.041599	2.18	FOXO6	forkhead box O6 [Source:HGNC Symbol;Acc:24814] [ENST00000372591]	
chr12:8072983-8072924	A_24_P202319	0.01937	2	ATP2A3	ATPase, Ca++ transporting, ubiquitous (ATP2A3), transcript variant 7, mRNA [NM_174958]	
chr12:9091964-9092023	A_32_P192970	0.013797	1.94	ALDH4A1	aldehyde dehydrogenase 4 family, member A1 (ALDH4A1), nuclear gene encoding mitochondrial protein, transcript variant P5CDhS, mRNA [NM_170726]	
chr12:124081285-124081226	A_23_P159741	0.042344	1.93	BCOR	BCL6 corepressor (BCOR), transcript variant 1, mRNA [NM_017745]	
chr12:119617464-119624868	A_19_P00319540	0.049533	1.91	XLOC_011183		
chr12:9093716-9093657	A_19_P00810009	0.047691	1.83	LOC100506948	PREDICTED: Homo sapiens hypothetical LOC100506948 (LOC100506948), miscRNA [XR_110299]	
chr12:27848353-27848412	A_19_P00322183	0.03099	1.81	XLOC_000515	BROAD Institute lincRNA (XLOC_000515), lincRNA [TCONS_00001233]	
chr12:59266361-59266302	A_24_P53519	0.030612	1.8	CHAF1A	chromatin assembly factor 1, subunit A (p150) (CHAF1A), mRNA [NM_005483]	
chr12:118501609-118501550	A_23_P31273	0.041228	1.75	AMPH	amphiphysin (AMPH), transcript variant 1, mRNA [NM_001635]	
chr12:121408137-121408078	A_33_P3334220	0.032932	1.68	ACACB	acetyl-CoA carboxylase beta (ACACB), mRNA [NM_001093]	
chr12:53346116-53346554	A_23_P204252	0.035462	1.62	M6PR	mannose-6-phosphate receptor (cation dependent) (M6PR), transcript variant 1, mRNA [NM_002355]	
chr12:47159136-47159077	A_33_P3784253	0.036802	1.55	PAK1	p21 protein (Cdc42/Rac)-activated kinase 1 (PAK1), transcript variant 1, mRNA [NM_001128620]	
chr12:19672916-19672975	A_33_P3228892	0.041077	1.5	LOC100130370	PREDICTED: Homo sapiens hypothetical protein	

					LOC100130370 (LOC100130370), mRNA [XM_001714096]	
chr12:57980041-57980100	A_33_P3373358	0.038331	1.5	GJC1	gap junction protein, gamma 1, 45 kDa (GJC1), transcript variant 1, mRNA [NM_005497]	
chr12:8482118-8482177	A_23_P256148	0.025116	1.49	AKIRIN1	akirin 1 (AKIRIN1), transcript variant 1, mRNA [NM_024595]	
chr12:102139417-102139358	A_33_P3618429	0.02375	1.47	PRKXP1	cDNA FLJ38474 fis, clone FEBRA2022255. [AK095793]	
chr12:8800746-8800687	A_24_P49214	0.049714	1.44	C1orf86	chromosome 1 open reading frame 86 (C1orf86), transcript variant 1, mRNA [NM_001146310]	
chr12:8077035-8075591	A_23_P131215	0.047301	1.44	CRYGD	crystallin, gamma D (CRYGD), mRNA [NM_006891]	
chr12:3149391-3149555	A_23_P15582	0.03507	1.43	XYLT2	xylosyltransferase II (XYLT2), mRNA [NM_022167]	
chr12:52180422-52180481	A_23_P213592	0.025724	1.42	RNF44	ring finger protein 44 (RNF44), mRNA [NM_014901]	
chr12:53294995-53294461	A_24_P577694	0.04483	1.41	ADCY1	adenylate cyclase 1 (brain) (ADCY1), mRNA [NM_021116]	
chr12:124421269-124421210	A_32_P70724	0.024163	1.39	KDM5B	lysine (K)-specific demethylase 5B (KDM5B), mRNA [NM_006618]	
chr12:99007538-99007479	A_33_P3293114	0.049608	1.25	SRSF11	serine/arginine-rich splicing factor 11 (SRSF11), transcript variant 2, mRNA [NM_001190987]	
chr12:10560299-10560240	A_21_P0011931	0.043236	1.21	MLL3	myeloid/lymphoid or mixed-lineage leukaemia 3 (MLL3), mRNA [NM_170606]	

References for EMT-related genes in Supplementary Table S10 and S11

1 Jiang, J., Liu, H.L., Liu, Z.H., Tan, S.W. & Wu, B. Identification of cystatin SN as a novel biomarker for pancreatic cancer. *Tumour Biol*. **36**, 3903-3910 (2015).

2 Riches, K. et al. Apolipoprotein(a) acts as a chemorepellent to human vascular smooth muscle cells via integrin alphaVbeta3 and RhoA/ROCK-mediated mechanisms. *Int. J. Biochem. Cell Biol.* **45**, 1776-1783 (2013).

3 Lee, C. C. et al. Association of acute phase protein-haptoglobin, and epithelial-mesenchymal transition in buccal cancer: a preliminary report. *Clin. Chem. Lab. Med.* **51**, 429-437 (2013).

4 Zhao, M., Kong, L., Liu, Y. & Qu, H. dbEMT: an epithelial-mesenchymal transition associated gene resource. *Sci. Rep.* **5**, 11459 (2015).

5 Chen, Y. H., Ishii, M., Sucov, H. M. & Maxson, R. E., Jr. Msx1 and Msx2 are required for endothelial-mesenchymal transformation of the atrioventricular cushions and patterning of the atrioventricular myocardium. *BMC Dev, Biol.* **8**, 75 (2008).

6 Gururajan, M. et al. miR-154* and miR-379 in the DLK1-DIO3 microRNA mega-cluster regulate epithelial to mesenchymal transition and bone metastasis of prostate cancer. *Clin. Cancer Res.* **20**, 6559-6569 (2014).

7 Fujiwara, H. et al. Regulation of mesodermal differentiation of mouse embryonic stem cells by basement membranes. *J. Biol. Chem.* **282**, 29701-29711 (2007).

8 Wang, X. et al. S100A14, a mediator of epithelial-mesenchymal transition, regulates proliferation, migration and invasion of human cervical cancer cells. *Am. J. Cancer Res.* 5, 1484-1495 (2015).

9 Zhang, X. et al. Cytosolic TMEM88 promotes invasion and metastasis in lung cancer cells by binding DVLS. *Cancer Res*. (2015).

10 Wang, H. et al. A systematic approach identifies FOXA1 as a key factor in the loss of epithelial traits during the epithelial-to-mesenchymal transition in lung cancer. *BMC Genomics* **14**, 680 (2013).

11 Pacurari, M. et al. The microRNA-200 family targets multiple non-small cell lung cancer prognostic markers in H1299 cells and BEAS-2B cells. *Int. J. Oncol.* **43**, 548-560 (2013).

12 Kim, H. et al. Correlation between PDZK1, Cdc37, Akt and breast cancer malignancy: the role of PDZK1 in cell growth through Akt stabilization by increasing and interacting with Cdc37. *Mol. Med*. **20**, 270-279 (2014).

13 Yu, T. et al. Effects of nicotinamide N-methyltransferase on PANC-1 cells proliferation, metastatic potential and survival under metabolic stress. *Cell Physiol. Biochem.* **35**, 710-721 (2015).

14 Hildebrand, A. et al. Interaction of the small interstitial proteoglycans biglycan, decorin and fibromodulin with transforming growth factor beta. *Biochem. J.* **302** (Pt 2), 527-534 (1994).

15 Wu, Y. C., Tang, S. J., Sun, G. H. & Sun, K. H. CXCR7 mediates TGFbeta1-promoted EMT and tumor-initiating features in lung cancer. *Oncogene* (2015).

16 Plouhinec, J. L. et al. Pax3 and Zic1 trigger the early neural crest gene regulatory network by the direct activation of multiple key neural crest specifiers. *Dev. Biol.* **386**, 461-472 (2014).

17 López-Lago, M. A. et al. Genomic deregulation during renal cell carcinoma metastasis implements a myofibroblast-like gene expression program. *Cancer Res.* **70**, 9682-9692 (2010).

18 Berta, J. et al. Apelin promotes lymphangiogenesis and lymph node metastasis. *Oncotarget* **5**, 4426-4437 (2014).

19 Leung, C.S. et al. Calcium-dependent FAK/CREB/TNNC1 signalling mediates the effect of stromal MFAP5 on ovarian cancer metastatic potential. *Nat. Commun.* **5**, 5092 (2014).

20 Lamouille, S., Xu, J. & Derynck, R. Molecular mechanisms of epithelial–mesenchymal transition. *Nat. Rev. Mol. Cell Biol.* **15**, 178-196 (2014).

21 Huttlin, E. L. et al. The BioPlex Network: a systematic exploration of the human interactome. *Cell* **162**, 425-440 (2015).

22 Shankar, J. et al. Pseudopodial actin dynamics control epithelial-mesenchymal transition in metastatic cancer cells. *Cancer Res*. **70**, 3780-3790 (2010).

23 Clay, M. R. & Halloran, M. C. Cadherin 6 promotes neural crest cell detachment via F-actin regulation and influences active Rho distribution during epithelial-to-mesenchymal transition. *Development* **141**, 2506-2515 (2014).

24 Vittal, R. et al. IL-17 induces type V collagen overexpression and EMT via TGF-beta-dependent pathways in obliterative bronchiolitis. *Am. J. Physiol. Lung Cell Mol. Physiol.* **304**, L401-L414 (2013).

25 Thomson, S. et al. A systems view of epithelial–mesenchymal transition signaling states. *Clin. Exp. Metastasis* **28**, 137-155 (2011).

26 Izumi, K. et al. Targeting the androgen receptor with siRNA promotes prostate cancer metastasis through enhanced macrophage recruitment via CCL2/CCR2-induced STAT3 activation. *EMBO Mol. Med.* **5**, 1383-1401 (2013).

27 Warrier, S. et al. Cancer stem-like cells from head and neck cancers are chemosensitized by the Wnt antagonist, sFRP4, by inducing apoptosis, decreasing stemness, drug resistance and epithelial to mesenchymal transition. *Cancer Gene Ther.* **21**, 381-388 (2014).

28 Natsuizaka, M. et al. Insulin-like growth factor-binding protein-3 promotes transforming growth factor-{beta}1-mediated epithelial-to-mesenchymal transition and motility in transformed human esophageal cells. *Carcinogenesis* **31**, 1344-1353 (2010).
29 Wang, X. et al. Epithelial neoplasia coincides with exacerbated injury and fibrotic response in the lungs of Gprc5a-knockout mice following silica exposure. *Oncotarget* (2015).
30 Pei, X. H., Lv, X. Q. & Li, H. X. Sox5 induces epithelial to mesenchymal transition by transactivation of Twist1. *Biochem. Biophys. Res. Commun.* **446**, 322-327 (2014).
31 Cojoc, M. et al. Aldehyde dehydrogenase is regulated by beta-Catenin/TCF and promotes radioresistance in prostate cancer progenitor cells. *Cancer Res.* **75**, 1482-1494 (2015).
32 Asanoma, K. et al. Regulation mechanism of TWIST1 transcription by BHLHE40 and BHLHE41 in cancer cells. *Mol. Cell. Biol*. (2015).
33 Volakis, L. I. et al. Loss of myoferlin redirects breast cancer cell motility towards collective migration. *PLoS One* **9**, e86110 (2014).
34 Zafar, A. et al. Chromatinized protein kinase C-theta directly regulates inducible genes in epithelial to mesenchymal transition and breast cancer stem cells. *Mol. Cell Biol.* **34**, 2961-29680 (2014).
35 Filant, J., Lydon, J. P. & Spencer, T. E. Integrated chromatin immunoprecipitation sequencing and microarray analysis identifies FOXA2 target genes in the glands of the mouse uterus. *FASEB J.* **28**, 230-243 (2014).
36 Gupta, S. et al. FZD4 as a mediator of ERG oncogene-induced WNT signalling and epithelial-to-mesenchymal transition in human prostate cancer cells. *Cancer Res*. **70**, 6735-6745 (2010).
37 Rivera-Feliciano, J. et al. Development of heart valves requires Gata4 expression in endothelial-derived cells. *Development* **133**, 3607-3618 (2006).
38 Guan, F., Schaffer, L., Handa, K. & Hakomori, S.I. Functional role of gangliotetraosylceramide in epithelial-to-mesenchymal transition process induced by hypoxia and by TGF-{beta}. *FASEB J.* **24**, 4889-4903 (2010).
39 Zhou, C. et al. Proteomic analysis of tumor necrosis factor-alpha resistant human breast cancer cells reveals a MEK5/Erk5-mediated epithelial-mesenchymal transition phenotype. *Breast Cancer Res*. **10**, R105 (2008).
40 Margetts, P. J. et al. Transient overexpression of TGF-{beta}1 induces epithelial mesenchymal transition in the rodent peritoneum. *J. Am. Soc. Nephrol.* **16**, 425-436 (2005).
41 Lehner, A. et al. Downregulation of serine protease HTRA1 is associated with poor survival in breast cancer. *PLoS One* **8**, e60359 (2013).
42 Yu, C. C. et al. Bmi-1 regulates snail expression and promotes metastasis ability in head and neck squamous cancer-derived ALDH1 positive cells. *J. Oncol.* **2011**, pii: 609259 (2011).

43 Konecny, G. E. et al. Expression of p16 and retinoblastoma determines response to CDK4/6 inhibition in ovarian cancer. *Clin. Cancer Res.* **17**, 1591-1602 (2011).
44 Gardi, N. L., Deshpande, T. U., Kamble, S. C., Budhe, S. R. & Bapat, S. A. Discrete molecular classes of ovarian cancer suggestive of unique mechanisms of transformation and metastases. *Clin. Cancer Res*. **20**, 87-99 (2014).
45 Read, M. L. et al. The proto-oncogene PBF binds p53 and is associated with prognostic features in colorectal cancer. *Mol. Carcinog.* (2014).
46 Josson, S. et al. miR-409-3p/-5p promotes tumorigenesis, epithelial-to-mesenchymal transition, and bone metastasis of human prostate cancer. *Clin. Cancer Res*. **20**, 4636-4646 (2014).
47 Fan, Y. et al. TGF-beta-induced upregulation of malat1 promotes bladder cancer metastasis by associating with suz12. *Clin. Cancer. Res*. **20**, 1531-1541 (2014).

Supplementary Table S12. H9 cell colonies demonstrating the highest correlation coefficients in cluster-A in the colony database

Colony numbers (ID No. in black: H9; ID No. in blue: Cluster-A)	1	188	2	193	3	159	4	188	5	201	6	159	7	183
Correlation coefficient	0.70		0.67		0.93		0.87		0.88		0.88		0.97	
Texture_Fourier_Descriptors_15	−0.26	−0.25	−0.31	−0.31	−0.34	−0.59	−0.11	−0.25	−0.35	−0.36	−0.57	−0.59	−0.43	−0.49
Texture_Fourier_Descriptors_10	0.19	−0.17	−0.20	−0.33	−0.33	−0.49	−0.27	−0.17	0.20	−0.42	−0.33	−0.49	−0.42	−0.60
Texture_Fourier_Descriptors_12	−0.17	−0.21	−0.35	−0.28	−0.27	−0.52	−0.05	−0.21	−0.15	−0.42	−0.41	−0.52	−0.26	−0.46
Texture_Fourier_Descriptors_0	−0.32	−0.46	0.14	−0.45	−0.11	−0.41	−0.46	−0.46	−0.49	−0.46	−0.30	−0.41	−0.46	−0.49
Texture_Fourier_Descriptors_19	0.26	−0.38	0.04	−0.48	−0.63	−0.60	0.47	−0.38	−0.22	−0.57	−0.52	−0.60	−0.34	−0.60
Texture_Fourier_Descriptors_18	−0.51	−0.44	0.29	−0.37	−0.48	−0.50	−0.09	−0.44	−0.36	−0.62	−0.47	−0.50	−0.55	−0.63
Texture_Fourier_Descriptors_6	0.64	−0.47	−0.01	−0.31	−0.57	−0.55	−0.21	−0.47	−0.33	−0.39	−0.49	−0.55	−0.45	−0.54
Texture_Fourier_Descriptors_3	−0.30	−0.37	−0.52	−0.37	−0.48	−0.51	0.07	−0.37	−0.31	−0.34	−0.53	−0.51	−0.28	−0.58
Texture_Fourier_Descriptors_1	−0.42	−0.66	0.65	0.24	−0.32	−0.64	−0.06	−0.66	0.31	−0.10	−0.26	−0.64	−0.42	−0.61
Texture_Fourier_Descriptors_11	−0.45	−0.54	−0.13	−0.13	−0.38	−0.51	0.30	−0.54	−0.19	−0.45	−0.65	−0.51	−0.40	−0.66
Texture_Fourier_Descriptors_16	−0.41	−0.31	−0.45	−0.19	−0.40	−0.43	−0.34	−0.31	0.04	−0.29	−0.41	−0.43	−0.38	−0.49
Texture_Fourier_Descript	−0.34	−0.66	−0.68	−0.36	−0.55	−0.62	−0.18	−0.66	−0.17	−0.56	−0.59	−0.62	−0.50	−0.51

ors_17														
Texture_Fourier_Descriptors_13	0.13	−0.59	−0.28	−0.36	−0.37	−0.45	−0.35	−0.59	0.18	−0.41	−0.28	−0.45	−0.50	−0.54
Texture_Fourier_Descriptors_14	−0.24	−0.22	−0.17	−0.49	−0.39	−0.61	−0.26	−0.22	−0.17	−0.28	−0.36	−0.61	−0.39	−0.49
Texture_Fourier_Descriptors_8	−0.13	−0.28	−0.32	−0.45	−0.49	−0.52	0.01	−0.28	−0.35	−0.32	−0.42	−0.52	−0.38	−0.54
Texture_Fourier_Descriptors_2	0.51	−0.27	−0.28	−0.36	−0.48	−0.62	−0.61	−0.27	−0.02	−0.36	−0.47	−0.62	−0.29	−0.55
Texture_Fourier_Descriptors_4	0.04	−0.24	−0.04	−0.23	−0.36	−0.52	0.33	−0.24	−0.29	−0.46	−0.40	−0.52	−0.37	−0.48
Texture_Fourier_Descriptors_9	−0.18	−0.43	0.48	−0.24	−0.52	−0.41	−0.48	−0.43	0.00	−0.37	−0.48	−0.41	−0.31	−0.51
Texture_Fourier_Descriptors_5	−0.37	−0.52	−0.45	−0.37	−0.59	−0.49	−0.48	−0.52	−0.42	−0.55	−0.45	−0.49	−0.18	−0.55
Texture_Fourier_Descriptors_7	−0.34	−0.32	0.07	−0.30	−0.50	−0.55	−0.15	−0.32	−0.34	−0.44	−0.51	−0.55	−0.36	−0.53
Shape_Shape_Factor	−1.02	−0.80	−0.95	−0.91	−1.02	−1.12	−1.15	−0.80	−1.17	−0.74	−0.67	−1.12	−1.40	−1.45
Shape_Equivalent_Radius	−0.58	−0.85	−0.04	−0.40	0.06	0.47	−1.69	−0.85	0.69	−0.11	−0.10	0.47	−0.84	−0.89
Volume_Area	−0.49	−0.62	−0.18	−0.40	−0.10	0.20	−0.87	−0.62	0.39	−0.22	−0.21	0.20	−0.61	−0.64
Volume_Perimeter	−0.14	−0.42	0.15	−0.10	0.27	0.65	−0.83	−0.42	0.86	−0.02	−0.05	0.65	−0.01	0.00
Shape_Compactness	0.57	0.22	0.45	0.38	0.57	0.81	0.88	0.22	0.93	0.15	0.08	0.81	1.88	2.20
Shape_Inner_Radius	−−0.52	−1.21	0.13	−0.44	−1.09	−1.27	−2.31	−1.21	0.55	−0.07	−0.72	−1.27	−0.32	−1.05
Shape_Rod_Like_Width	−1.17	−1.57	−0.60	−0.88	−0.52	−0.73	−2.93	−1.57	0.47	−0.08	−0.42	−0.73	−1.72	−1.96

Supplementary Table S13. Antibodies used in this study

Antibody	Catalogue number and supplier
anti-OCT3/4 (POU domain, class 5, transcription factor 1) rabbit IgG	H134, sc9081, Santa Cruz Biotechnology, Santa Cruz, CA, USA
anti-vimentin mouse IgG1	V6630, Sigma-Aldrich, St. Louis, MO, USA
Alexa Fluor 555 goat anti-rabbit IgG (H+L)	A-21428, Life Technologies, Carlsbad, CA, USA
Alexa Fluor 488 goat anti-rabbit IgG (H+L)	A-11008, Life Technologies, Carlsbad, CA, USA
Alexa Fluor 488 goat anti-mouse IgG (H+L)	A-11001, Life Technologies, Carlsbad, CA, USA

Supplementary References

1 Thomson, J. A. *et al.* Embryonic stem cell lines derived from human blastocysts. *Science* **282**, 1145-1147 (1998).

2 Takahashi, K. *et al.* Induction of pluripotent stem cells from adult human fibroblasts by defined factors. *Cell* **131**, 861-872 (2007).

3 Nakagawa, M. *et al.* Generation of induced pluripotent stem cells without Myc from mouse and human fibroblasts. *Nat. Biotechnol.* **26**, 101-106 (2008).

4 Nagata, S. *et al.* Efficient reprogramming of human and mouse primary extra-embryonic cells to pluripotent stem cells. *Genes Cells* **14**, 1395-1404 (2009).

5 Makino, H. *et al.* Mesenchymal to embryonic incomplete transition of human cells by chimeric OCT4/3 (POU5F1) with physiological co-activator EWS. *Exp. Cell Res.* **315**, 2727-2740 (2009).

6 Inamura, M. *et al.* Efficient generation of hepatoblasts from human ES cells and iPS cells by transient overexpression of homeobox gene HEX. *Mol. Ther.* **19**, 400-407 (2011).

7 Kinehara, M. *et al.* Protein kinase C regulates human pluripotent stem cell self-renewal. *PLoS One* **8**, e54122 (2013).

8 Mimura, S. *et al.* Synergistic effects of FGF-2 and Activin A on early neural differentiation of human pluripotent stem cells. *In Vitro Cell. Dev. Biol. Anim.* **51**, 769-75 (2015).

9 Chen, G. *et al.* Chemically defined conditions for human iPSC derivation and culture. *Nat. Methods* **8**, 424-429 (2011).

10 Suga, M., Kii, H., Niikura, K., Kiyota, Y. & Furue, M. K. Development of a monitoring method for nonlabeled human pluripotent stem cell growth by time-lapse image analysis. *Stem Cells Transl. Med.* **4**, 720-730 (2015).

11 Ohnuma, K. *et al.* Enzyme-free passage of human pluripotent stem cells by controlling divalent cations. *Sci. Rep.* **4**, 4646 (2014).

12 International Stem Cell Initiative. *et al.* Characterization of human embryonic stem cell lines by the International Stem Cell Initiative. *Nat. Biotechnol.* **25**, 803-816 (2007).

13 Bock, C. *et al.* Reference maps of human ES and iPS cell variation enable high-throughput characterization of pluripotent cell lines. *Cell* **144**, 439-452 (2011).

14 Jacobs, J. P., Jones, C. M. & Baille, J. P. Characteristics of a human diploid cell designated MRC-5. *Nature* **227**, 168-170 (1970).

Supplementary Figures

Supplementary Figure S1. Biological characterization of the hiPSC 201B7 cell line and its subclone 201B7-1A

A. Representative morphological image of 2 clones. Compared with the parental clone 201B7, the aberrant clone 201B7-1A (#12 trisomy) exhibited colonies with more irregular morphology.

B. Representative data from the flow cytometry analysis of 2 clones with corresponding marker protein expression (Tra-1-60, Tra-1-81, Tra-2-54, CD90, SSEA3 and SSEA4). TRA-1-60, TRA-2-54, TRA-1-81, CD90, SSEA-3 and SSEA4 expression was tested for each hiPSC line. A FACSCanto flow cytometer (Becton, Dickinson and Company, Franklin Lakes, NJ, USA) was used to acquire data. FlowJo (http://www.flowjo.com/) was used for data analyses.

C. Teratoma derived from 201B7 and 201B7-1A cells. Paraffin-embedded sections were stained using H&E. a, b: black arrows indicate endodermal-like cells. c, d: black arrows indicate chondrocyte-like cells. e, f: black arrows indicate neural-like cells.

D–G. FISH analysis of 201B7, 201B7-1A and 253G1-B1 cells and G-banding of 253G1-B1 cells.

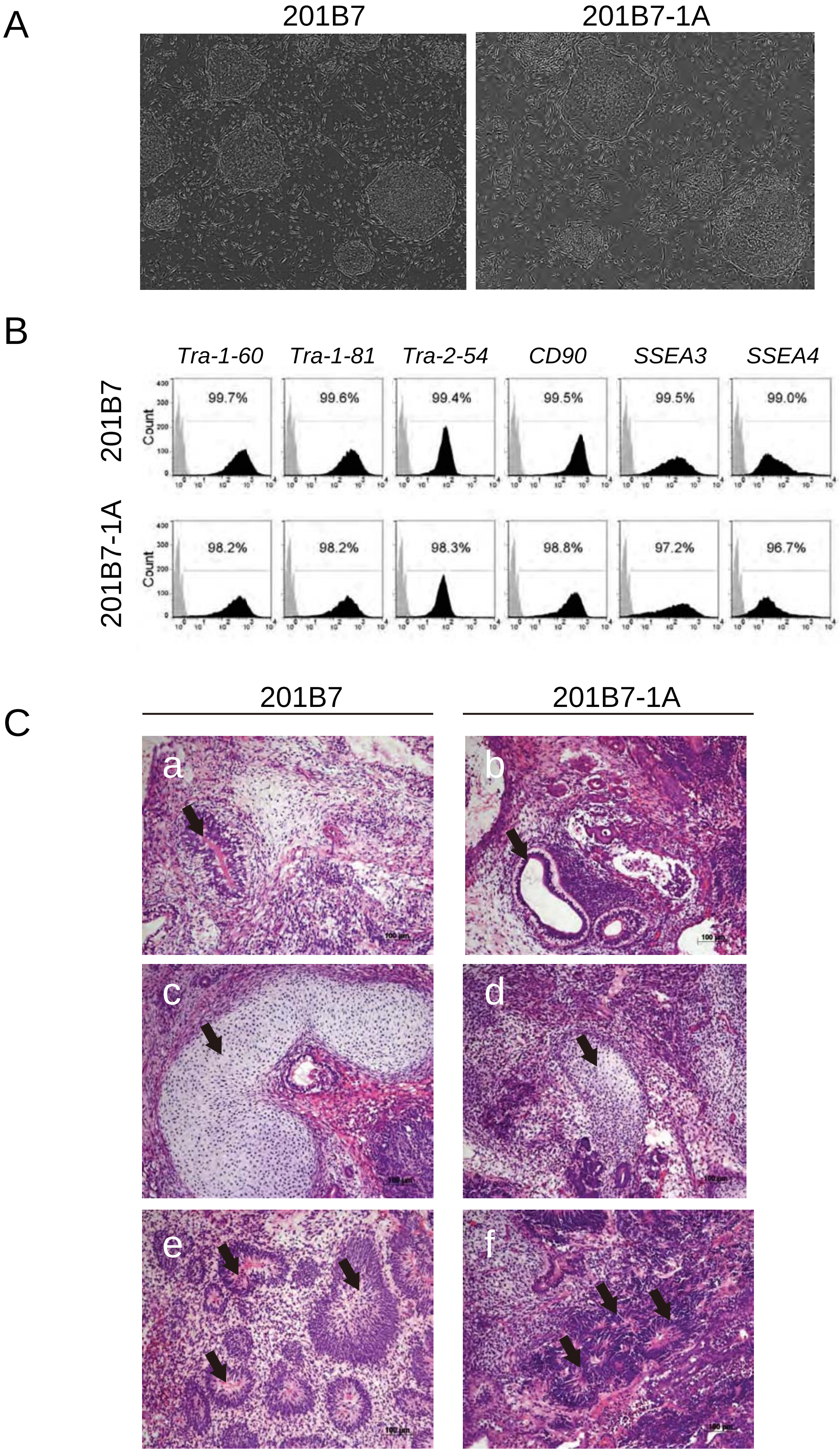

Supplementary Figure S1

201B7 P15+10

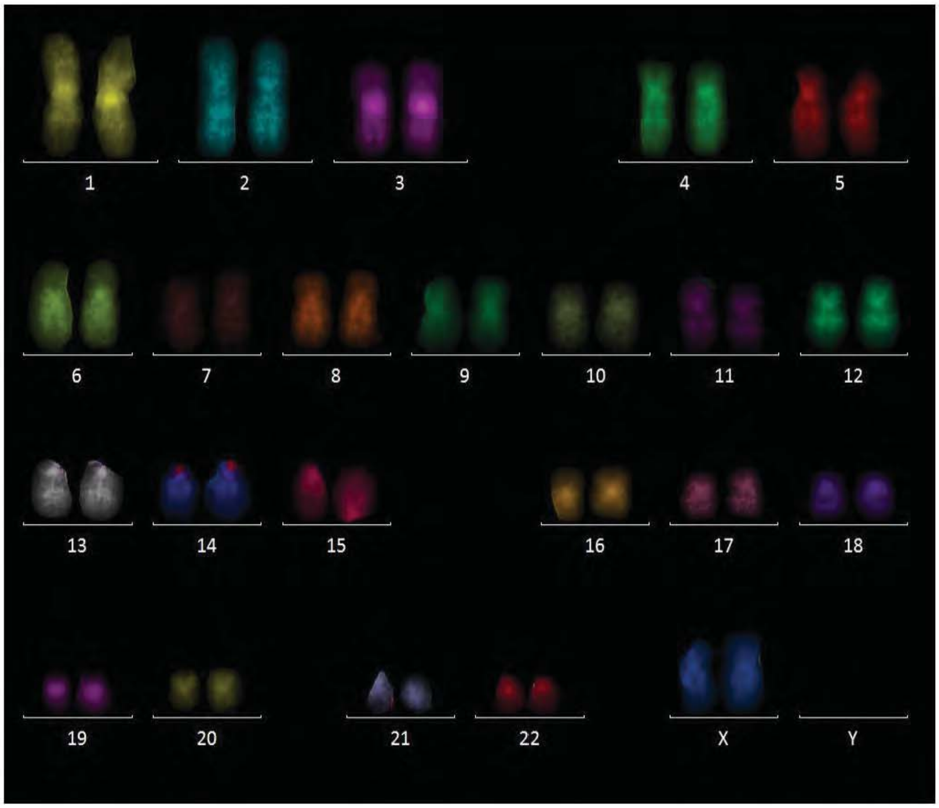

Supplementary Figure S1. D

201B7-1A P135+20

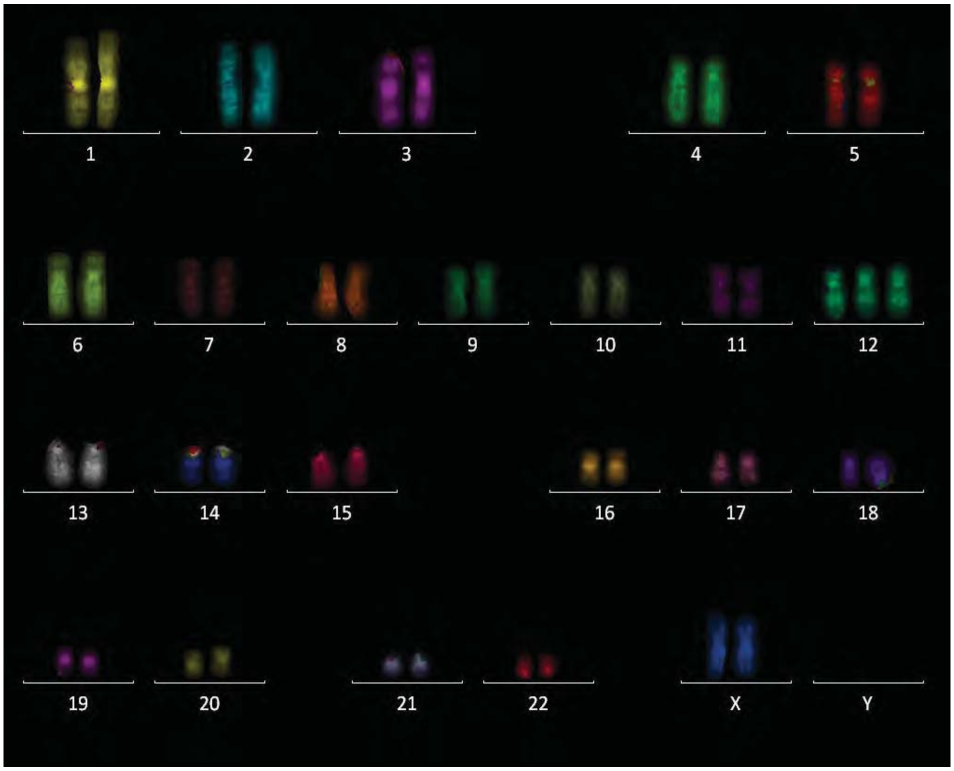

Supplementary Figure S1. E

253G1B p50+6

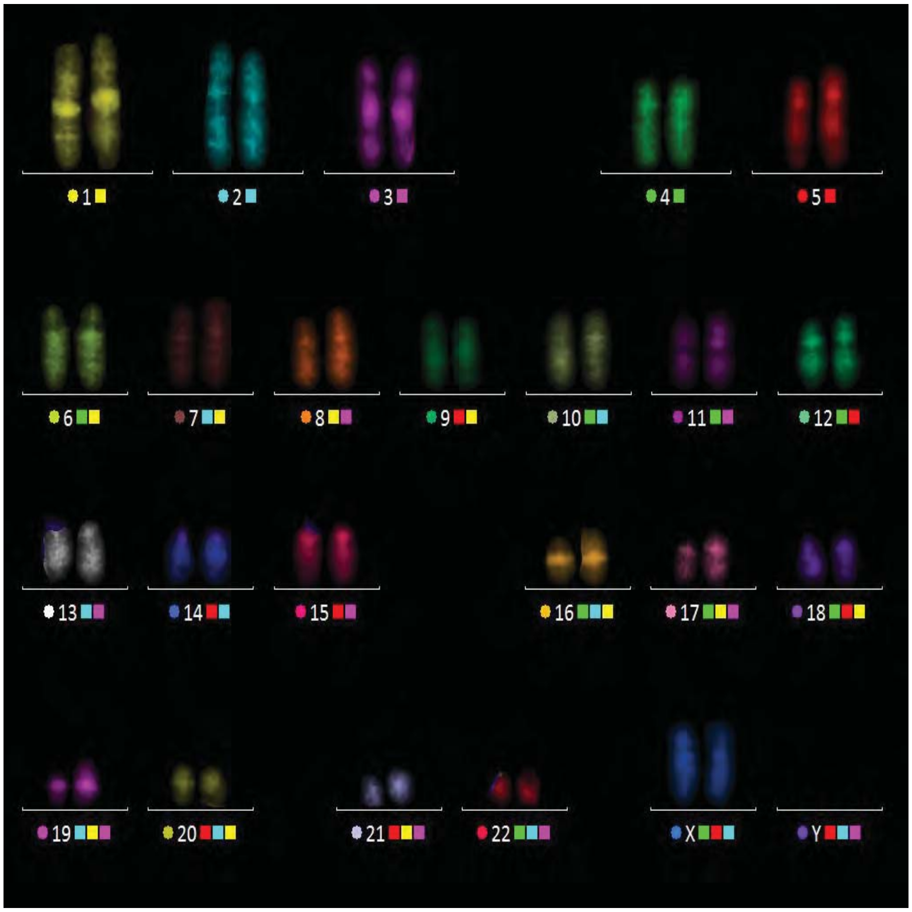

Supplementary Figure S1. F

253G1-B1 P5

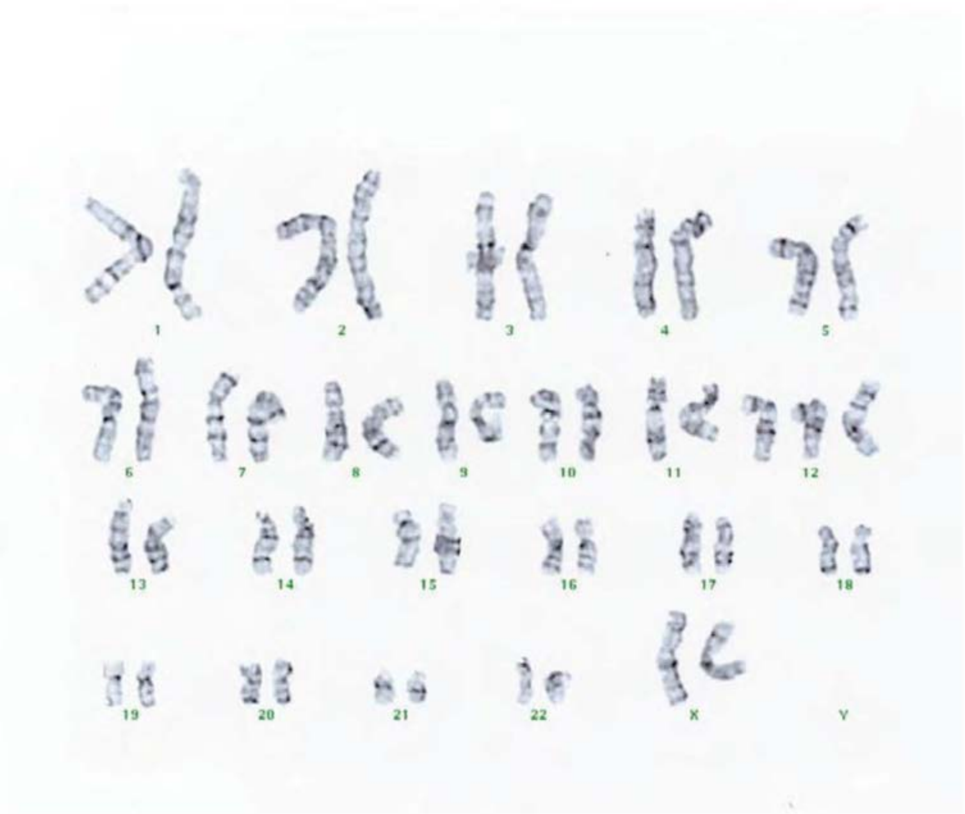

Supplementary Figure S1.G

Supplementary Figure S2. Schematic illustration of the morphological analysis of hPSC colonies in this study

Morphological analysis consisted of 2 parts. Step 1: Construction of the colony database. hPSCs were cultured in a 6-well plate for 6 days. 1-1: The colony image library was prepared by collecting colony phase contrast images every 8 h using an automatic live-cell imaging system. 1-2: The morphological parameters of the colonies in the image library were analysed. 1-3: Cluster analysis was performed to categorize colony morphologies. Step 2: Evaluation of live cultured colonies. Images of new 201B7 and 201B7-1A colonies cultured under the same protocol were captured, and morphological parameters were analysed. Colonies were classified into clusters by referring to the colony database constructed in Step 1.

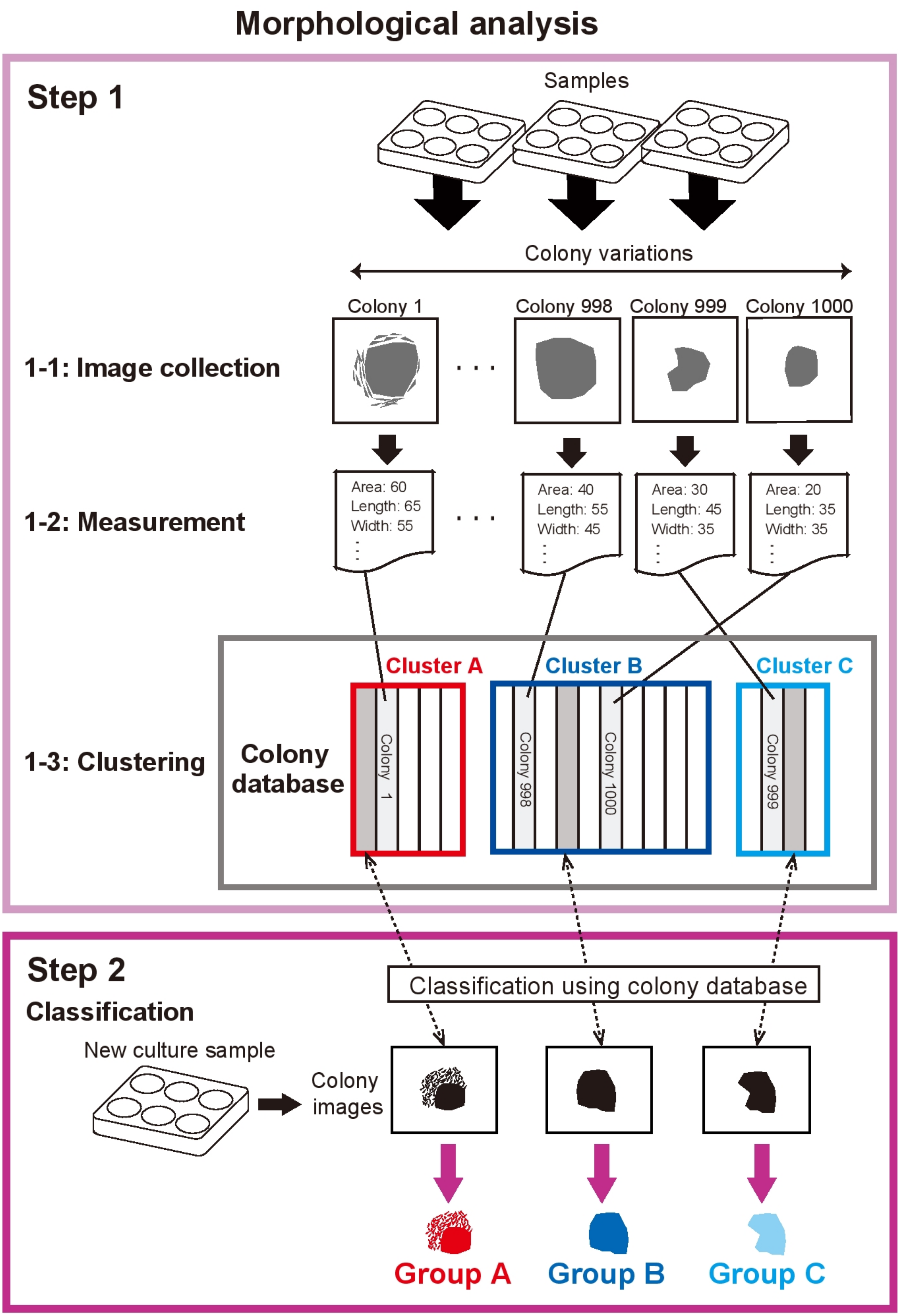

Supplementary Figure S2

Supplementary Figure S3. Adjustment of image acquisition

Tiled images at magnifications of 10×, 4× and 2× were compared to assess the clarity of the cellular details and the coverage size and speed. Magnification at 4× with 8 × 8 tiling provided the most stable, detailed and wide colony images covering 6-well plates containing cultured hiPSCs (resulting in the production of 1000 × 1000 $pixels^2$/image).

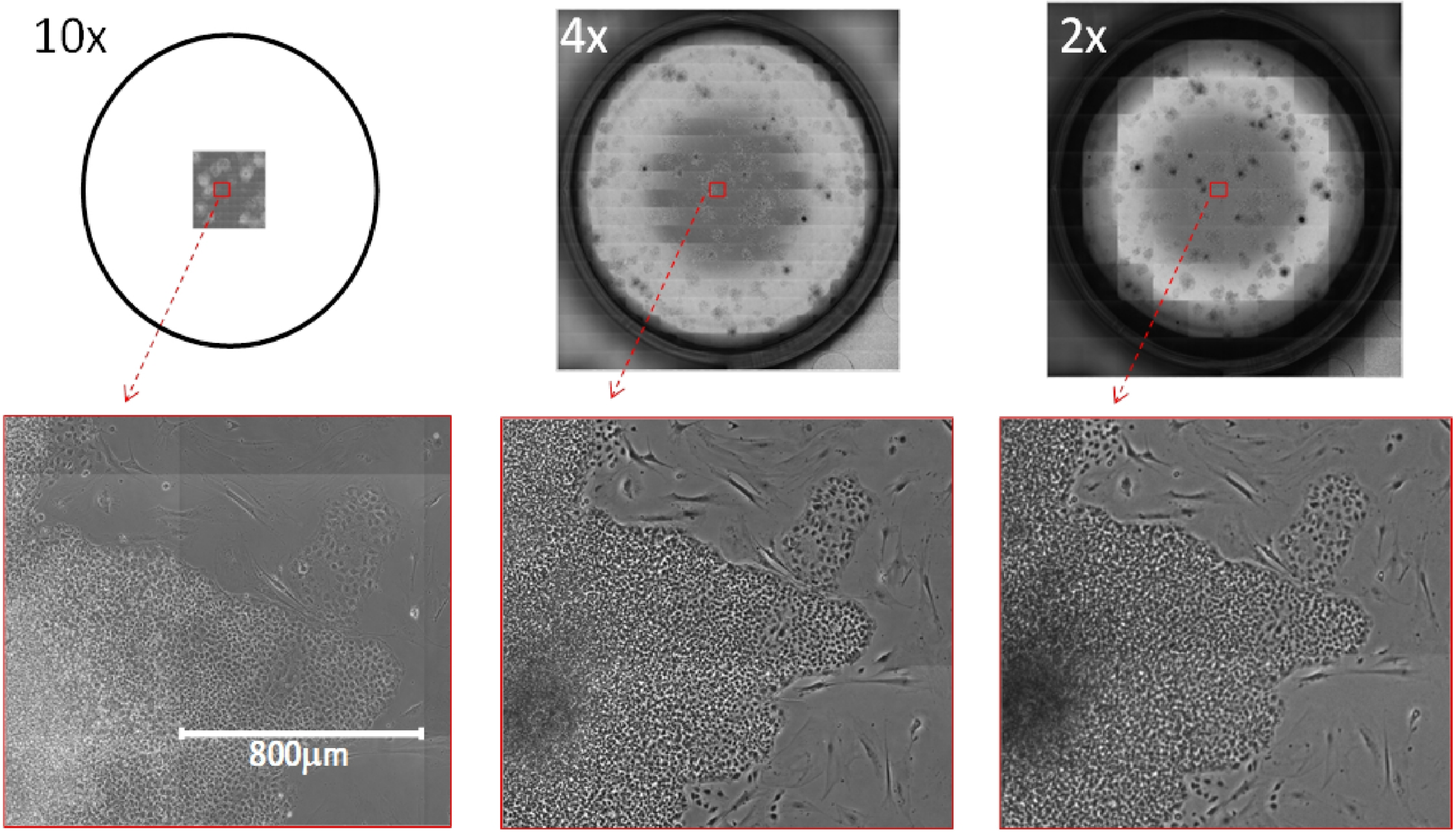

Supplementary Figure S3

Supplementary Figure S4. Representative morphologies of colonies classified into cluster-A/cluster-B/cluster-C/cluster-D/cluster-E

Three colony samples per morphological cluster are shown. In sample 1, pink (cluster-A) or green (other clusters) masks were overlaid to indicate colony recognition by our image processing scheme. Scale bar: 500 μm.

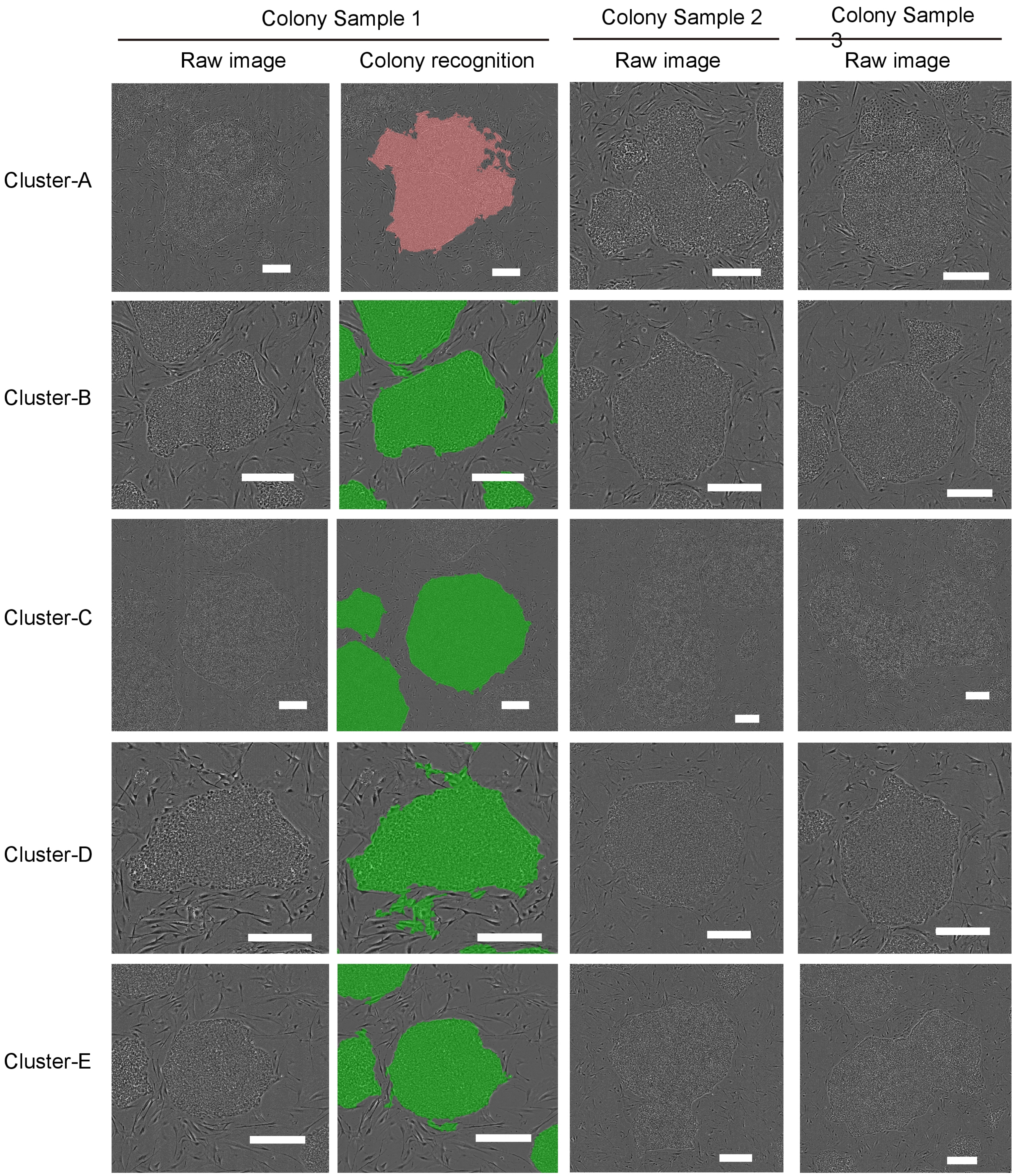

Supplementary Figure S4

Supplementary Figure S5. Reproducibility of morphological clusters following label masking of cell line names

The colony labels for 201B7, 201B7-1A, 253G1 and 253G1-B1 cells in the colony database were masked, and their data were re-clustered using our procedure. The clustering results are similar to those shown above, indicating the reproducibility of our method.

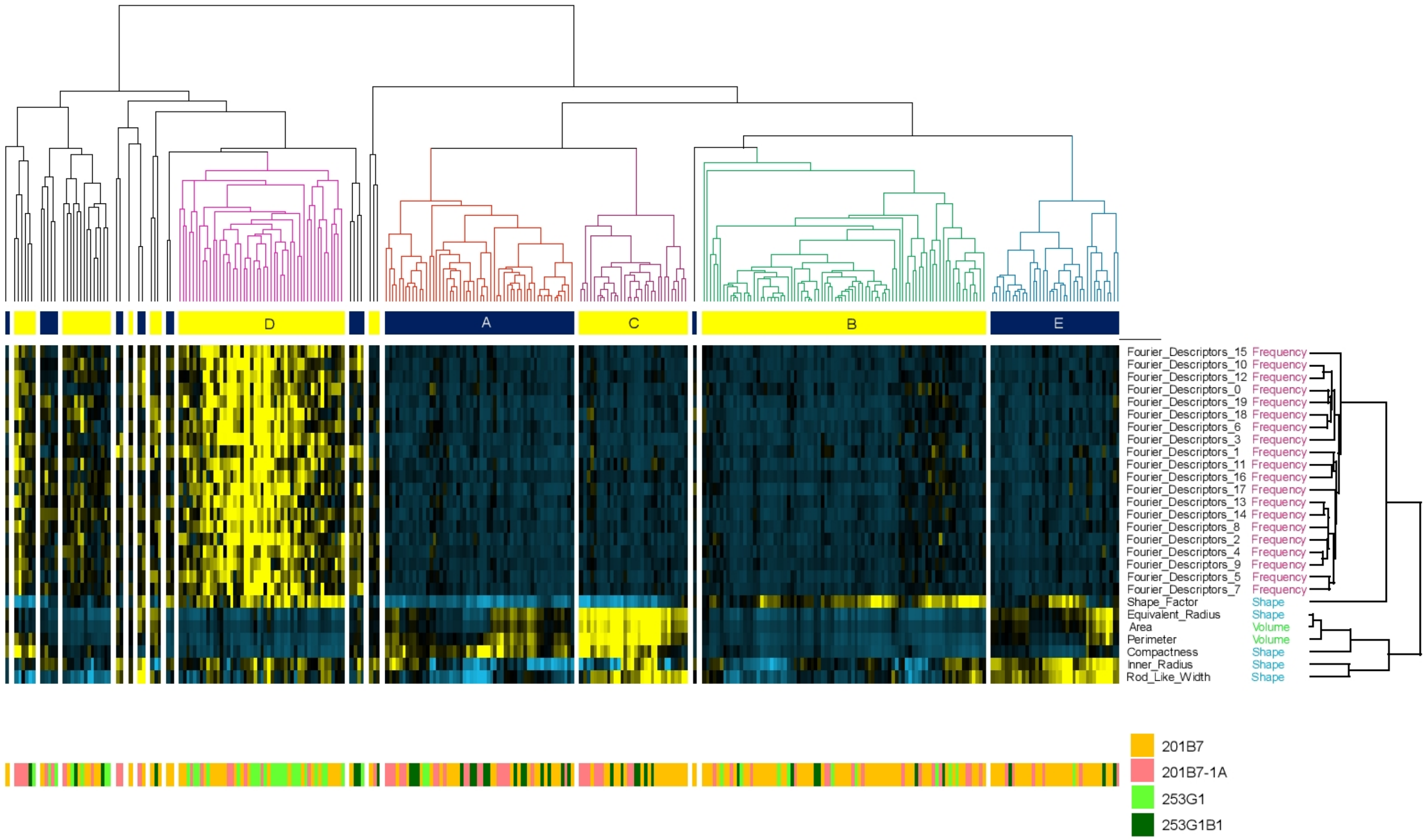

Supplementary Figure S5

Supplementary Figure S6. Representative colony recognition images from the rotated plates

Two representative colony recognition images that were obtained from the plate wells in their original position (0° degrees) and following physical rotation (180° degrees from the original position) are shown. The green region indicates the colony recognition area after image processing. Left: One of the 14 colonies classified into the same clusters is shown. Right: The recognition mask (green area) was unexpectedly different for one of the 15 colonies following plate rotation.

The white arrowheads indicate floating debris that crossed the colony edge following plate rotation. Due to the debris position, colony recognition was greatly altered. However, this mistake was found in only one of the 15 colonies analysed in the well.

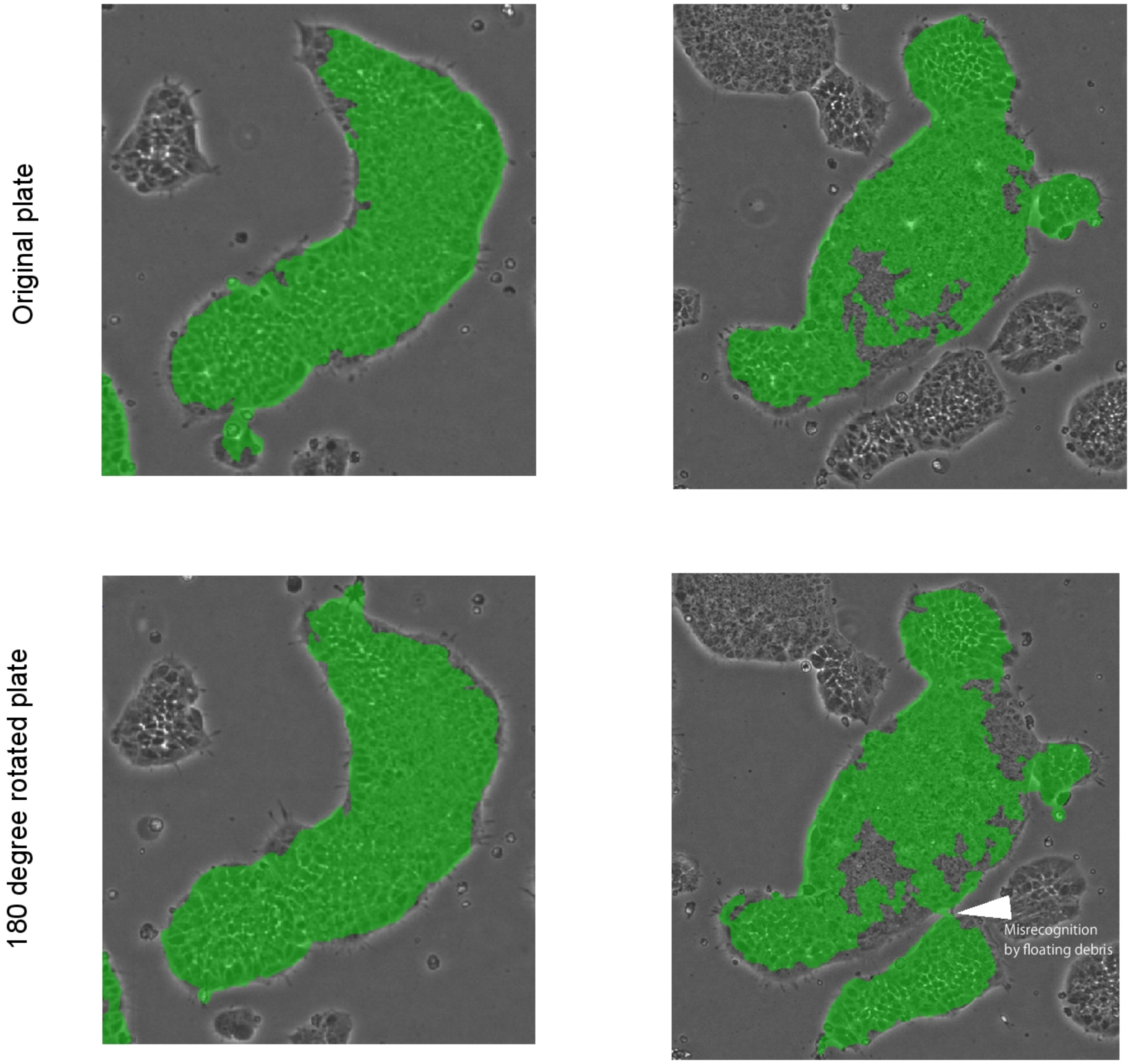

Supplementary Figure S6

Supplementary Figure S7. Overview of morphological varieties and colony morphology categories among Tic cells

An hiPSC line, Tic (P21+66), was cultured without feeder cells on vitronectin in TeSR-E8 medium. Standard Giemsa staining analysis revealed that Tic cells contained 46 chromosomes at P21+64. Tic cell images were captured using an automatic live-cell imaging system. The morphologies of 1009 Tic cell colonies were measured according to 27 morphological parameters and then underwent clustering analysis. Heat maps (blue = low, black = medium, yellow = high) show the normalized values of the 27 measured morphological parameters. The colonies were categorized into 19 clusters. There were 6 major morphological clusters (designated Major Tic 1–6), of which some were similar to the major clusters in the colony database; specifically, ‘Major Tic 3’ and ‘Major Tic 4’ were similar to cluster-B, and ‘Major Tic 6’ was similar to cluster-C.

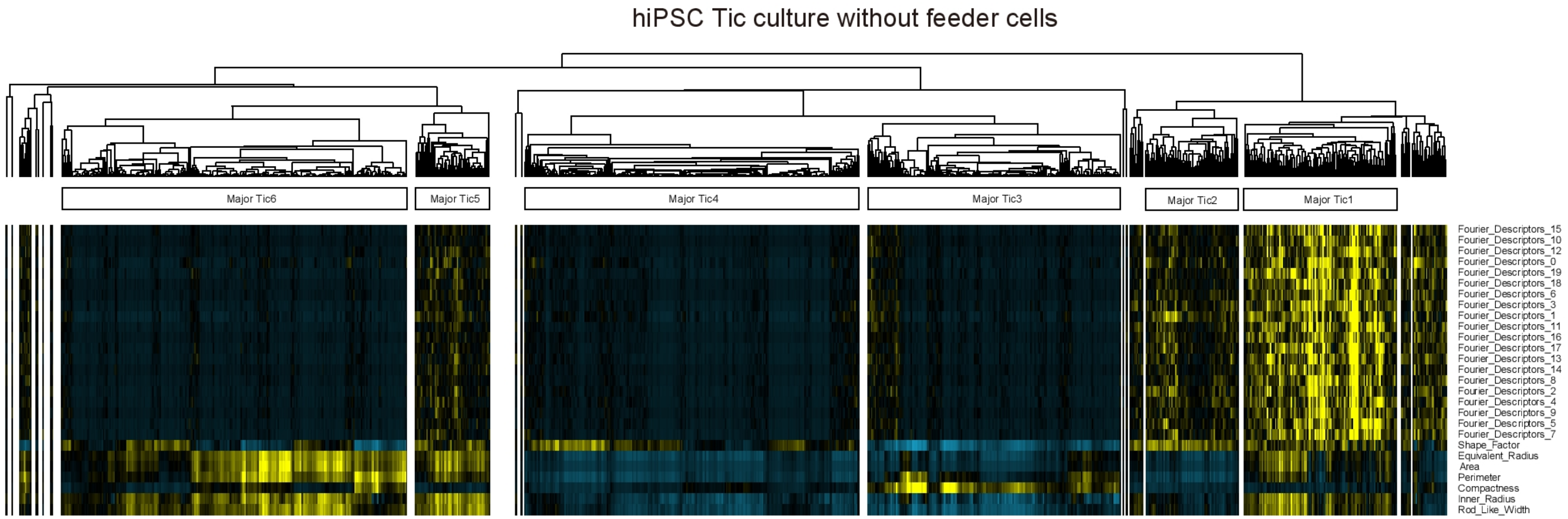

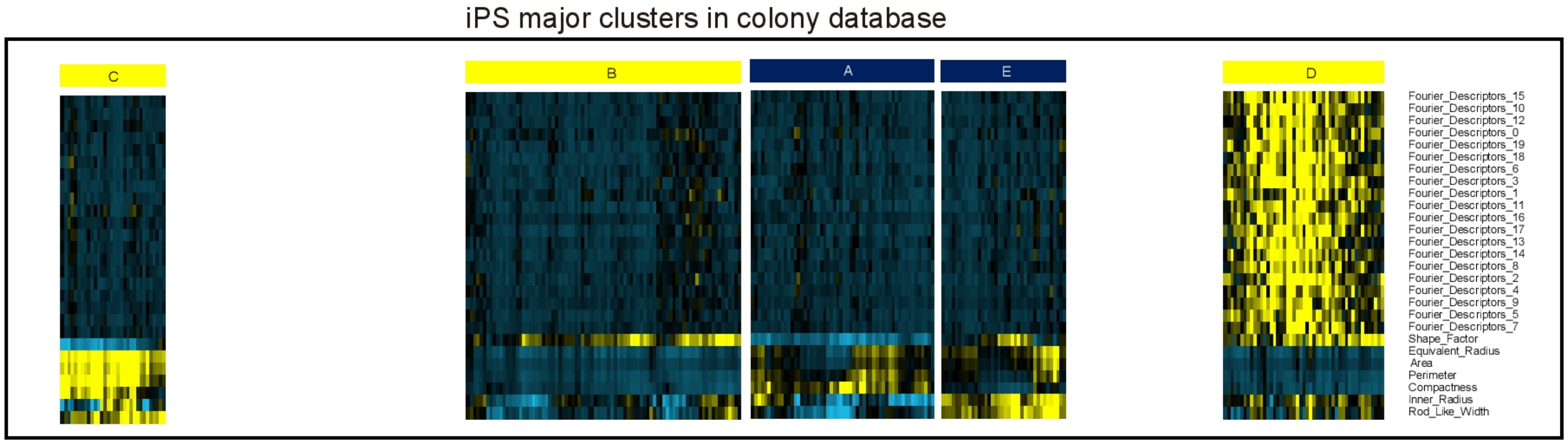

Supplementary Figure S7

Supplementary Figure S8. Representative morphology images of colonies (cluster-A, cluster-B, cluster-D, cluster-I, and cluster-J)

Representative images of colonies categorized into five morphological clusters are shown. (Top image) Colonies immunohistochemically stained with anti-OCT-3/4 (green) and anti-VIMENTIN antibodies (orange); (middle image) raw phase contrast image; (bottom image) image of the colony recognition mask following image processing for each cell type (201B7 or 201B7-1A). Scale bar = 500 µm. Among all colonies (cluster-A: 113 colonies, cluster-B: 332 colonies, cluster-D: 108 colonies, cluster-I: 41 colonies, and cluster-J: 10 colonies), those demonstrating high correlation (Pearson's correlation coefficient >0.8) were cropped for image representation. In 201B7 cells, cluster-A rarely appeared; therefore, only two highly correlated colonies are shown.

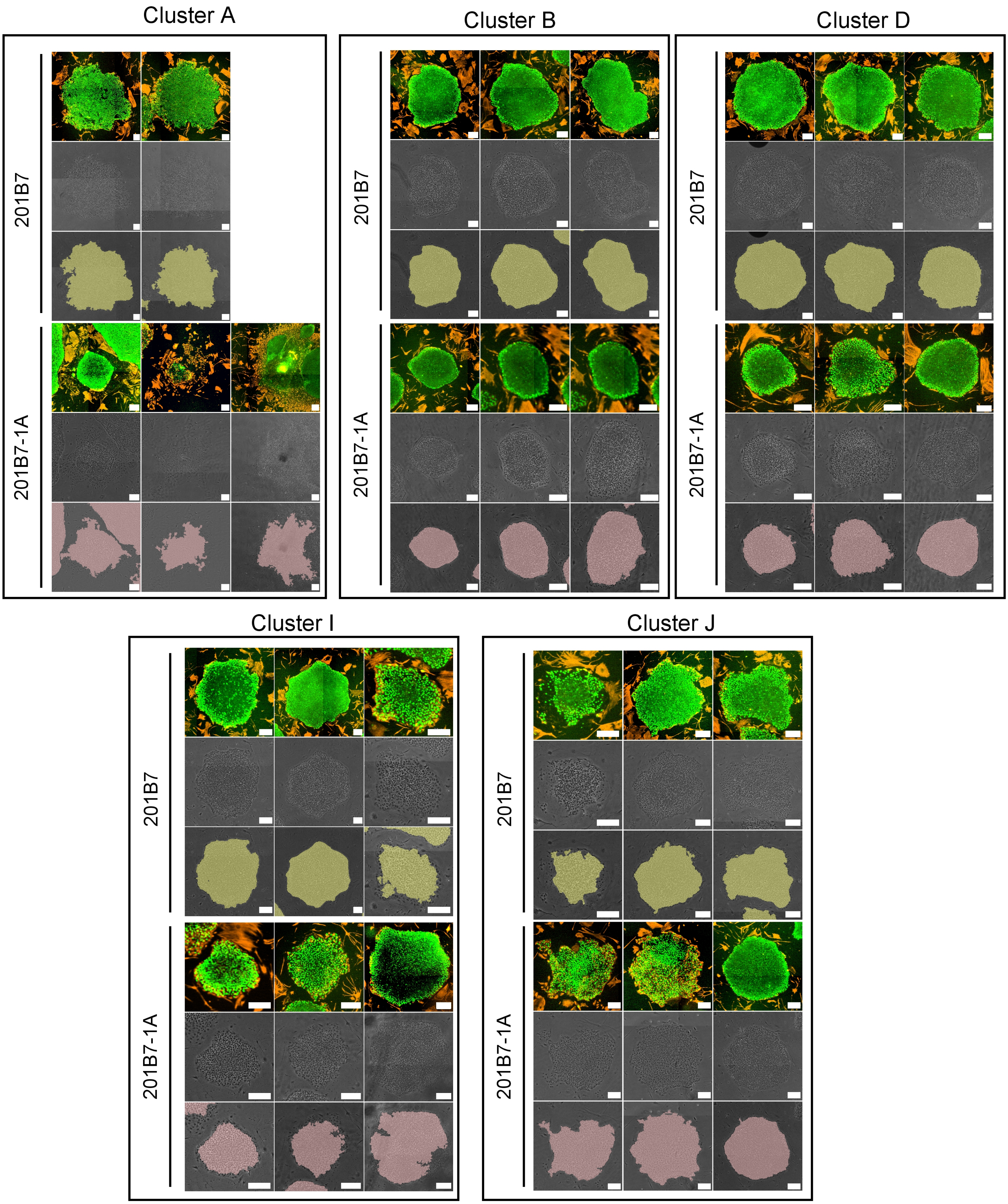

Supplementary Figure S9

Supplementary Figure S9. Schematic illustration of the correlations between colony morphological signature and biological characterization (gene expression profiles)

Independent of colony database construction or colony marker staining, 201B7 and 201B7-1A cells were freshly cultured and classified into clusters by referencing the constructed colony database. Colonies identified as target clusters (cluster-A/-B/-C is illustrated as an example) were directly selected for total mRNA extraction followed by global gene expression microarray analysis. Colonies analysed based on 'morphological parameters' or 'global gene expression profiles' were compared to understand the correlations between colony morphological signature and characteristic gene expression.

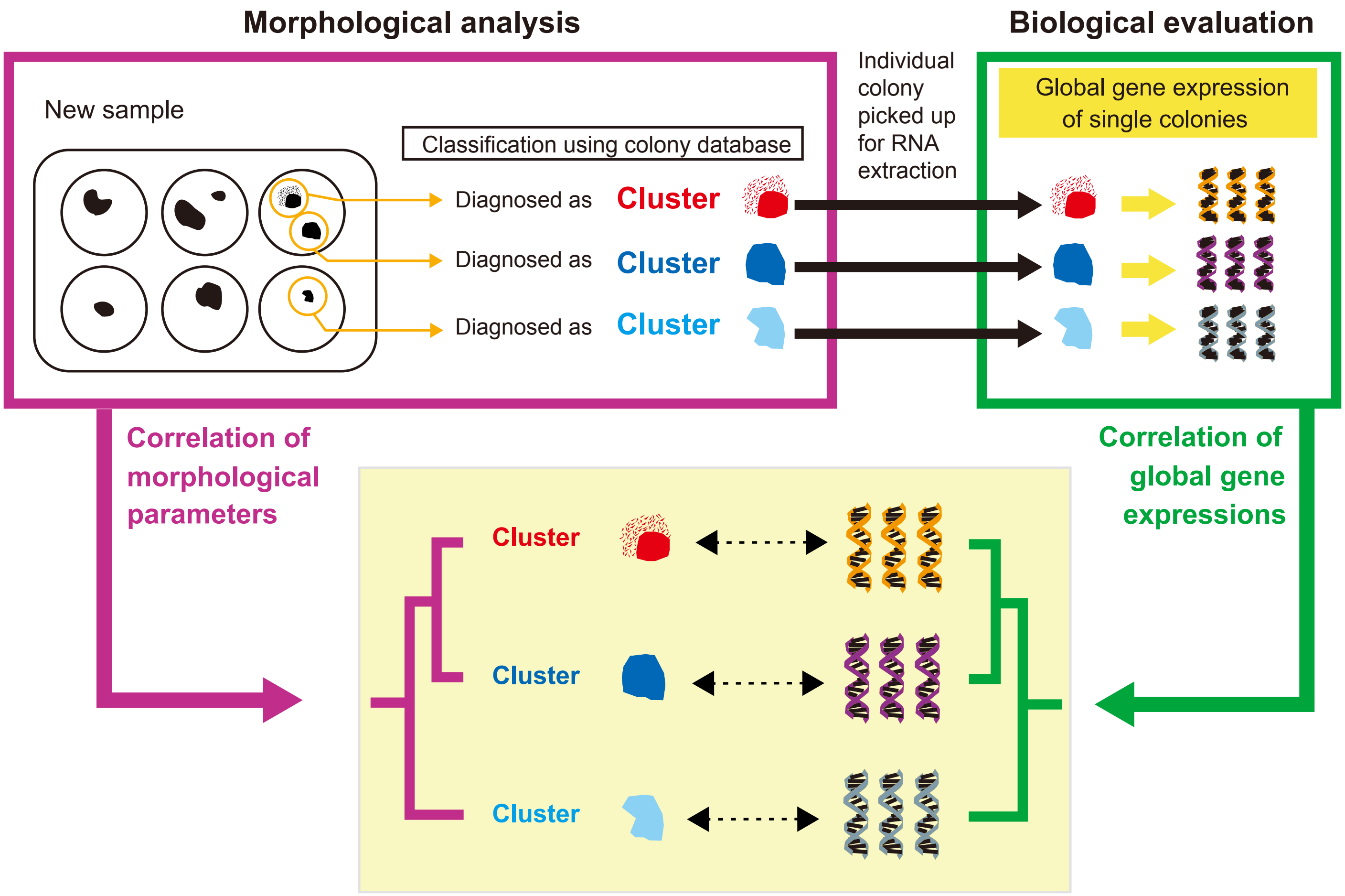

Supplementary Figure S9

Supplementary Figure S10. Images and colony recognition masks of selected colonies for whole-genome expression analysis

Green masks: masks recognizing colonies in 201B7. Pink masks: masks recognising colonies in 201B7-1A. The numbers in the images represent colony ID numbers. Boxed numbers represent colonies classified into cluster-A. Scale bar: 500 µm.

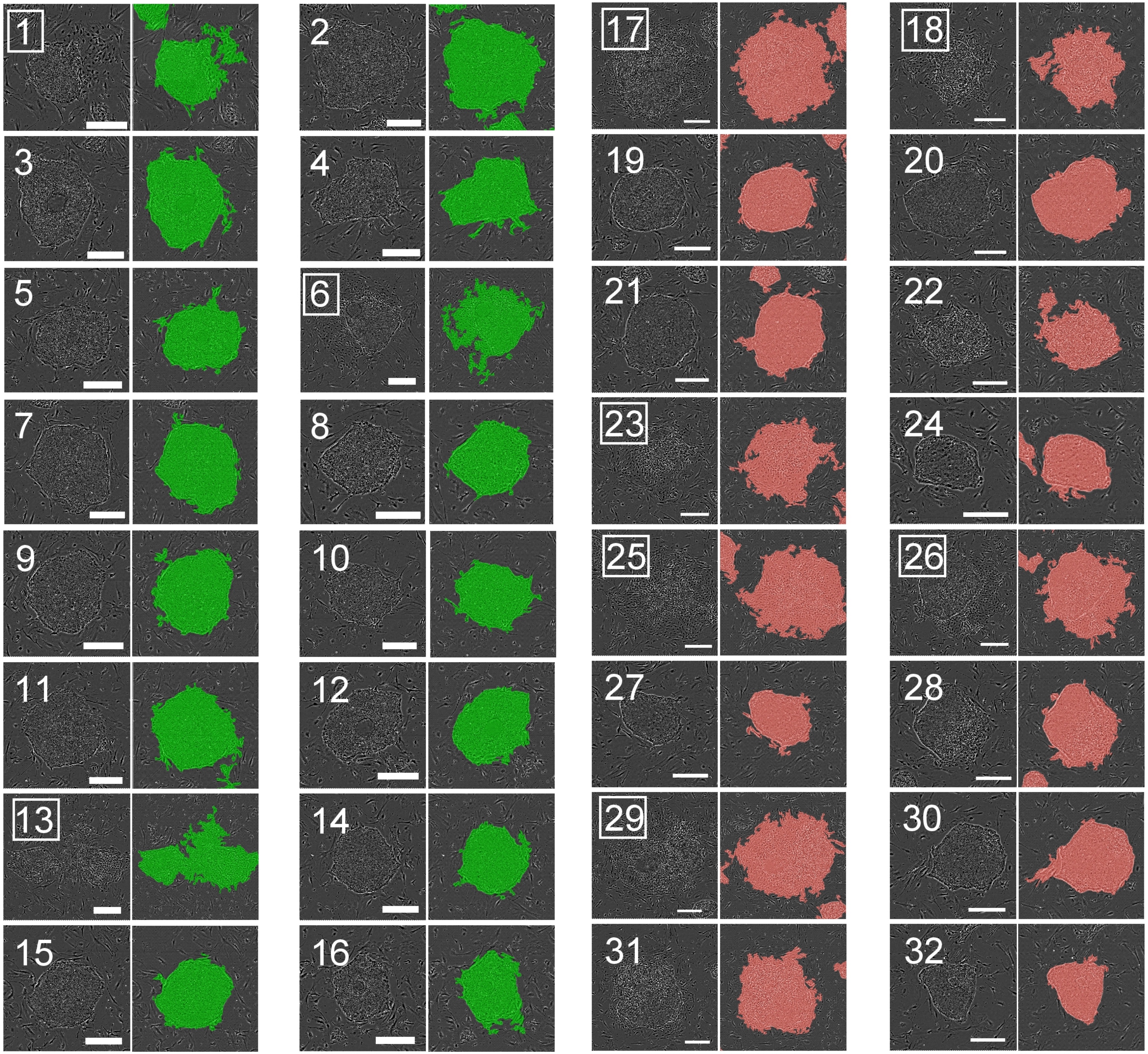

Supplementary Figure S10

Supplementary Figure S11. Overview of colony morphological varieties and categories in H9 cells

Images of an hESC line, H9 (P26+12), cultured on MEFs in KSR-based medium were captured using an automatic live-cell imaging system. Standard Giemsa staining analysis indicated H9 cells contain 46 chromosomes at P26+16. The morphologies of 537 H9 colonies were measured according to 27 morphological parameters and then underwent cluster analysis. Heat maps (blue = low, black = medium, yellow = high) indicate the normalized values of the 27 measured morphological parameters. The colonies were categorized into 39 clusters. There were 4 major morphological clusters (designated Major ES1–4), some of which were similar to the major clusters in the colony database; specifically, cluster ‘Major ES1’ was similar to cluster-B, and ‘Major ES3’ was similar to cluster-C. Blue, pink and yellow bars indicate the 3 corresponding wells containing cultured colonies.

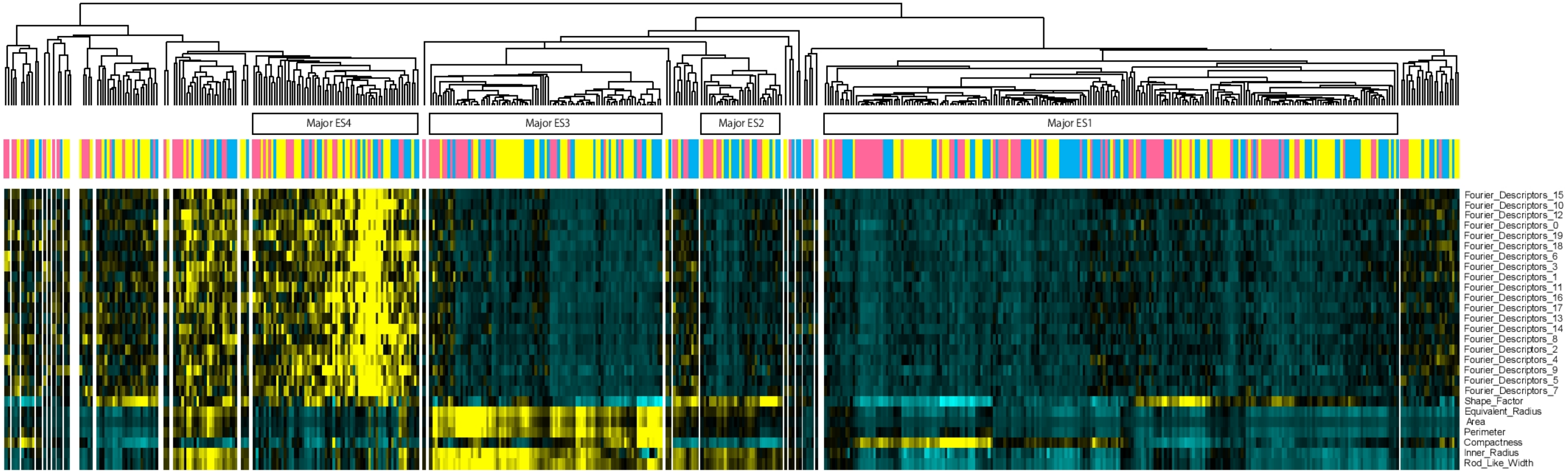

iPS major clusters in colony database

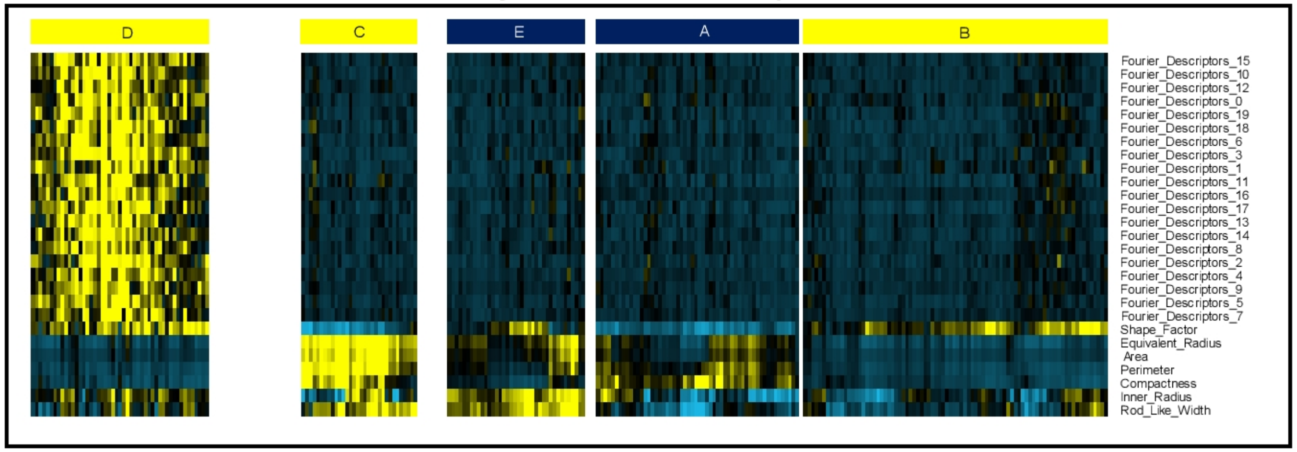

Supplementary Figure S11

Supplementary Figure S12. Images of H9 colonies classified into cluster-A

Phase-contrast raw images of H9 colonies classified into cluster-A. Cluster-A colonies exhibited irregular morphology with collapsed edges. Scale bar: 500 µm.

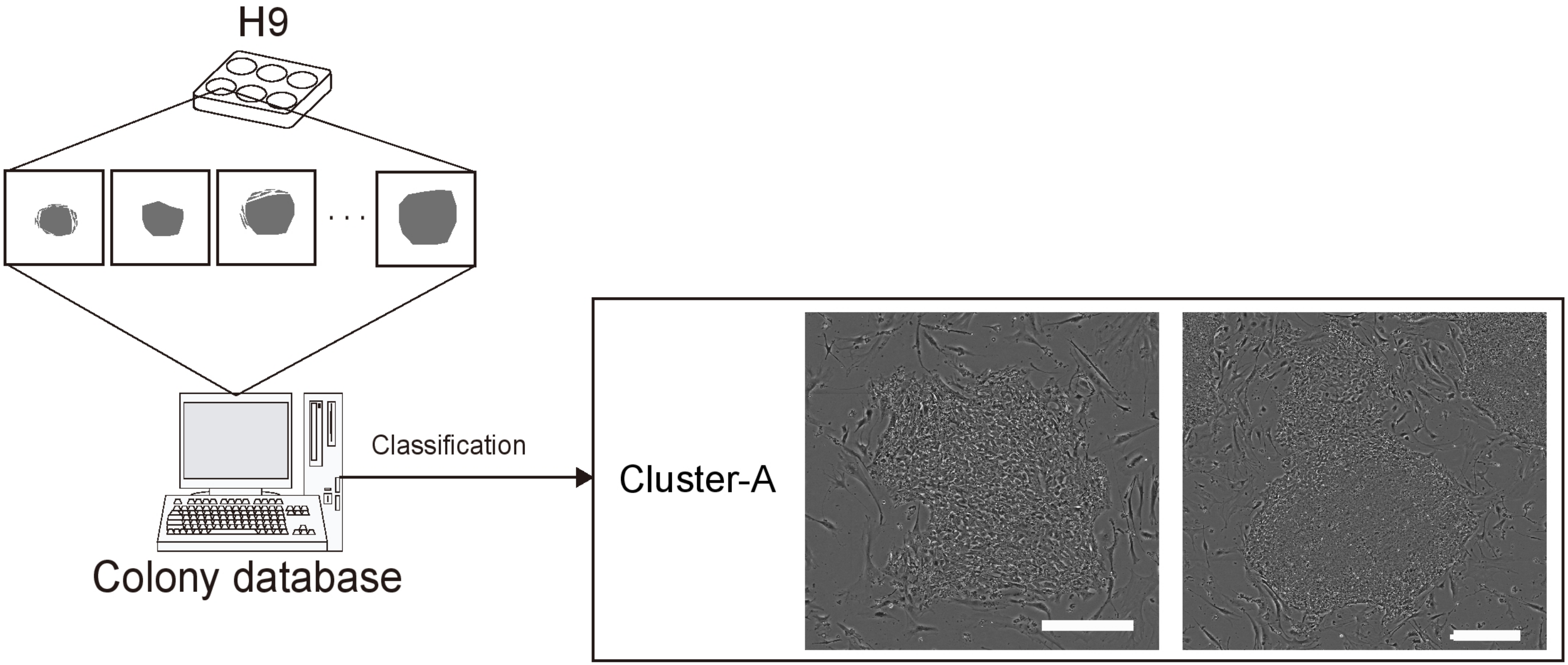

Supplementary Figure S12

Supplementary Figure S13. Schematic depicting the full image processing procedure

Colony recognition image processing was achieved using the procedure shown in this schematic, according to the instructions of the image analysis software. For each procedure, the cropped representative image (tiled image) and its image processing masks (green area) are shown.

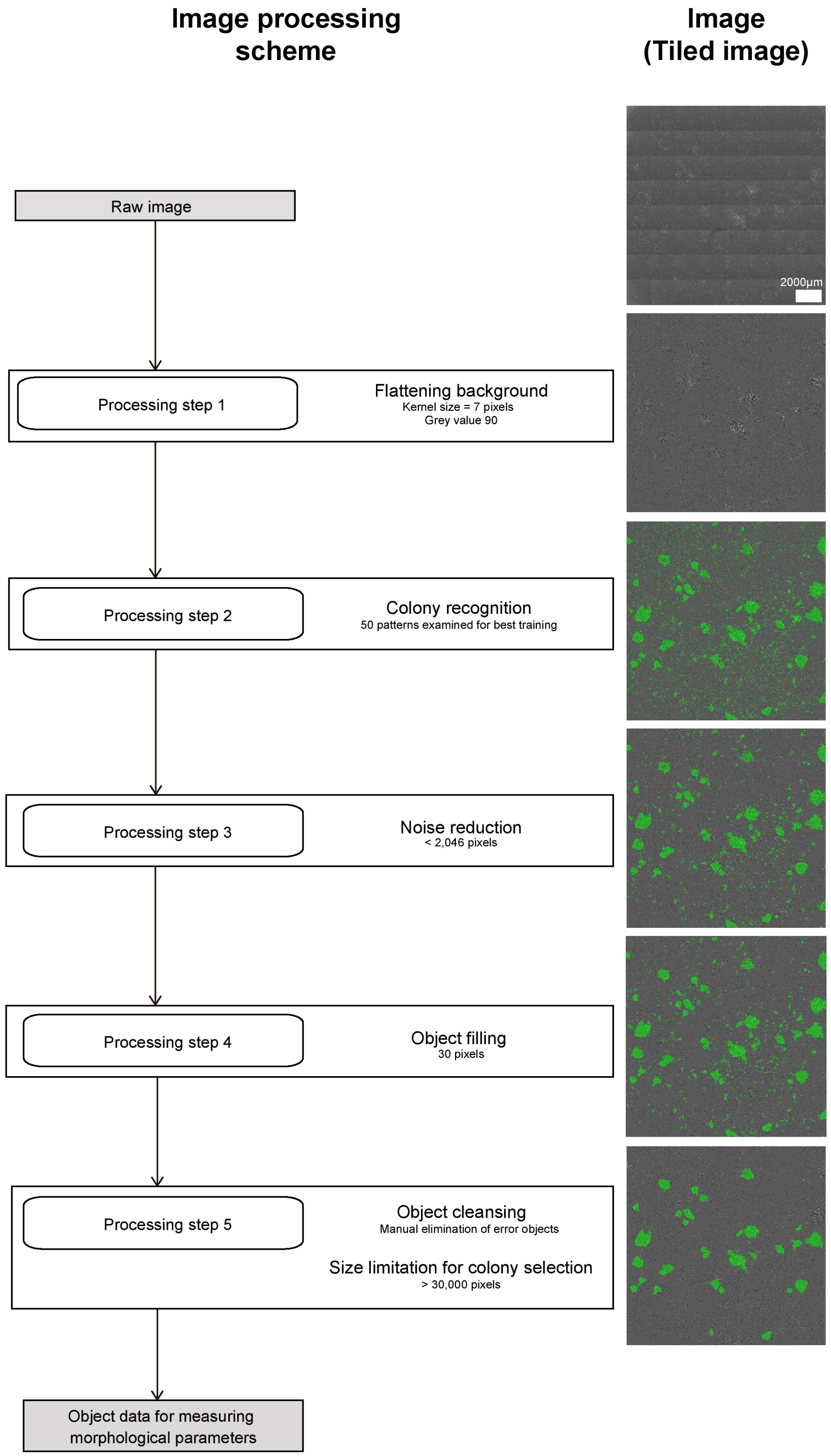

Supplementary Figure S13

Supplementary Figure S14. Schematic depicting the image processing procedure to identify representative cluster colonies

Colony recognition image processing (image processing step 1 to step 5) is shown with corresponding processed images (cropped images) of two sample colonies within each representative morphological cluster (cluster-A, -B, -D, -I, and -J).

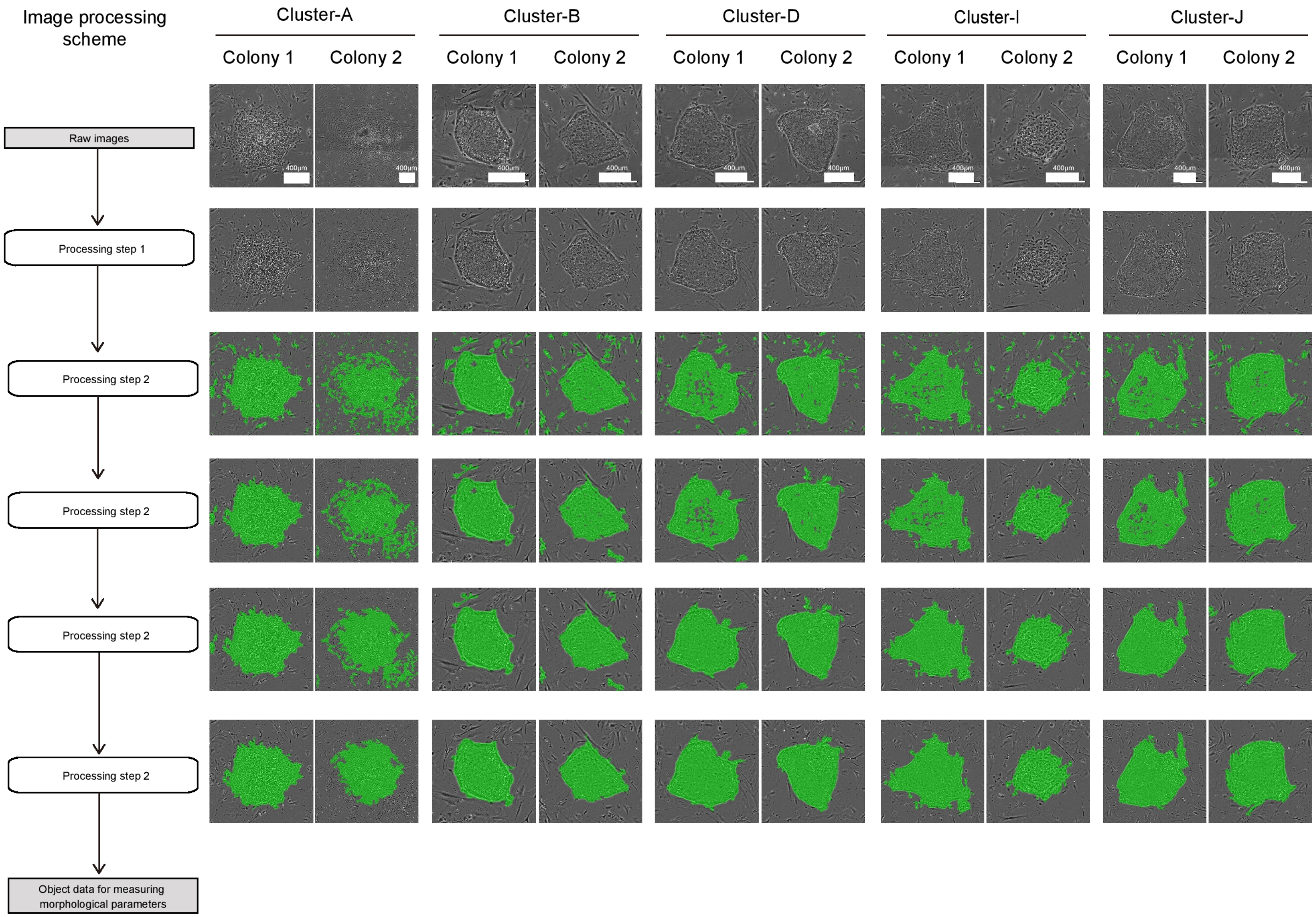

Supplementary Figure S14

Supplementary Videos

Supplementary Video S1

Time course video of morphological changes in 201B7. (Top video) Raw phase contrast microscopic images; (bottom video) coloured regions on raw images indicate the colony recognition area after image processing.

Supplementary Video S2

Time course video of morphological changes in 201B7-1A. (Top video) Raw phase contrast microscopic images; (bottom video) coloured regions on raw images indicate the colony recognition area after image processing.
